# Genetic diversity within the genus *Francisella* as revealed by comparative analyses of the genomes of two North American isolates from environmental sources

**DOI:** 10.1186/1471-2164-13-422

**Published:** 2012-08-24

**Authors:** Shivakumara Siddaramappa, Jean F Challacombe, Jeannine M Petersen, Segaran Pillai, Cheryl R Kuske

**Affiliations:** 1 Bioscience Division, Los Alamos National Laboratory, Los Alamos, NewMexico, 87545, USA; 2 Division of Vector-Borne Infectious Diseases,Centers for Disease Control and Prevention, Fort Collins, Colorado, 80521, USA; 3 Chemical and Biological Division, Science and Technology Directorate,Department of Homeland Security, Washington, DC, USA

## Abstract

**Background:**

*Francisella tularensis* is an intracellular pathogen that causes tularemia in humans and the public health importance of this bacterium has been well documented in recent history. *Francisella philomiragia*, a distant relative of *F*. *tularensis*, is thought to constitute an *environmental lineage* along with *Francisella novicida*. Nevertheless, both *F*. *philomiragia* and *F*. *novicida* have been associated with human disease, primarily in immune-compromised individuals. To understand the genetic relationships and evolutionary contexts among different lineages within the genus *Francisella*, the genome of *Francisella* spp. strain TX07-7308 was sequenced and compared to the genomes of *F. philomiragia* strains ATCC 25017 and 25015, *F*. *novicida* strain U112, and *F*. *tularensis* strain Schu S4.

**Results:**

The size of strain ATCC 25017 chromosome was 2,045,775 bp and contained 1,983 protein-coding genes. The size of strain TX07-7308 chromosome was 2,035,931 bp and contained 1,980 protein-coding genes. Pairwise BLAST comparisons indicated that strains TX07-7308 and ATCC 25017 contained 1,700 protein coding genes in common. NUCmer analyses revealed that the chromosomes of strains TX07-7308 and ATCC 25017 were mostly collinear except for a few gaps, translocations, and/or inversions. Using the genome sequence data and comparative analyses with other members of the genus *Francisella* (*e.g.*, *F*. *novicida* strain U112 and *F*. *tularensis* strain Schu S4), several strain-specific genes were identified. Strains TX07-7308 and ATCC 25017 contained an operon with six open reading frames encoding proteins related to enzymes involved in thiamine biosynthesis that was absent in *F*. *novicida* strain U112 and *F*. *tularensis* strain Schu S4. Strain ATCC 25017 contained an operon putatively involved in lactose metabolism that was absent in strain TX07-7308, *F*. *novicida* strain U112, and *F*. *tularensis* strain Schu S4. In contrast, strain TX07-7308 contained an operon putatively involved in glucuronate metabolism that was absent in the genomes of strain ATCC 25017, *F*. *novicida* strain U112, and *F*. *tularensis* strain Schu S4. The polymorphic nature of polysaccharide biosynthesis/modification gene clusters among different *Francisella* strains was also evident from genome analyses.

**Conclusions:**

From genome comparisons, it appeared that genes encoding novel functions have contributed to the metabolic enrichment of the *environmental lineages* within the genus *Francisella*. The inability to acquire new genes coupled with the loss of ancestral traits and the consequent reductive evolution may be a cause for, as well as an effect of, niche selection of *F. tularensis*. Sequencing and comparison of the genomes of more isolates are required to obtain further insights into the ecology and evolution of different species within the genus *Francisella*.

## Background

A small gram-negative bacterium that resembled members of the genus *Pasteurella* in many features was isolated in 1959 from a moribund muskrat found on the shoreline of a marsh on the Bear River Migratory Bird Refuge in northern Utah [1]. Based on morphological and biochemical characteristics, this bacterium was originally classified as *Yersinia philomiragia* (ATCC strain 25015^T^). Additional isolations (ATCC strains 25016, 25017, and 25018) of bacteria similar to the muskrat pathogen were made in 1960 from surface water samples collected in marshy areas within few miles of the Bear River Research Station, Utah [1]. Subsequent studies indicated that ATCC strains 25015^T^, 25016, 25017, and 25018 were biochemically and genetically different from members of the genus *Yersinia*[2]. Between 1975 and 1987, fourteen isolates resembling *Yersinia philomiragia* were reported from clinical cases [3]. These human isolates and the 4 ATCC strains from Utah were reclassified based on biochemical characteristics, cellular fatty acid compositions, and DNA hybridization studies as *Francisella philomiragia* (*philomiragia* = loving mirages, because of the mirages seen in the area where the first strains were found in Utah) in 1989 [4].

Most of the clinical isolates of *F. philomiragia* are from human cases associated with saltwater exposure and the first *environmental* isolates were from a marshy area that forms part of the Great Salt Lake waterway [3,4]. Not surprisingly, *F. philomiragia* is halotolerant and grows in media containing 6% NaCl, whereas *Francisella tularensis* does not [5]. Furthermore, in the laboratory, *F. philomiragia* is less fastidious than *F. tularensis* and does not require cysteine for growth [4]. *F. philomiragia* is considered an opportunistic pathogen and is most commonly isolated from patients with chronic granulomatous disease or those who experienced near-drowning in saltwater [3,4]. In contrast, *F. tularensis* is a highly infectious zoonotic agent and tularemia in healthy individuals exposed to contaminated freshwater sources has been reported [6]. Nevertheless, *F. tularensis* is generally not associated with saltwater exposure and is not known to cause near-drowning-associated pneumonia [7]. Isolations of *F. philomiragia* in the clinical laboratory have been sporadic and only four human cases have been reported in the last two decades [8-11]. A case of septicemia due to *F. philomiragia* has also been reported in a North Carolina dog that lived in a coastal town [12].

Recent attempts at direct isolation of *Francisella* spp. from environmental samples using cysteine heart agar with 9% chocolatized sheep blood have yielded three strains (TX07-6608, TX07-7308, and TX07-7310) from seawater collected in the Galveston Bay area, Texas [13]. Comparisons of 16S rRNA and *sdhA* genes from these strains with those of other *Francisella* spp. (*e.g.*, *F*. *novicida* strain U112 and *F*. *tularensis* strain Schu S4) have indicated that strain TX07-7308 is more similar to *F. philomiragia* than to *F. tularensis*[13]. A phylogenetic study based on 16S rRNA genes of several members of *Francisellaceae* has clustered strain TX07-7308 with an uncultured bacterium clone SSW64Au obtained from the Salton Sea in California [14]. In addition, *F. philomiragia* has been detected in samples obtained from a brackish-water pond in Martha’s Vineyard, Massachusetts [15]. Furthermore, a close taxonomic relationship between *F. philomiragia* and *Francisella noatunensis*, an emerging fish pathogen associated with marine environments, has also been established [16].

*Francisella novicida* is thought to constitute an *environmental lineage* along with *F. philomiragia*, and the genome of *F. novicida* strain U112 has been sequenced and compared to the genome sequences of *F. tularensis* strains pathogenic to humans [17]. The genomes of *F. novicida*-like strains Fx1 and 3523, which are clinical isolates from the USA and Australia, respectively, have also been sequenced and compared to the genome sequence of strain U112 [18]. In view of the ecological significance of *F. philomiragia*, the complete genome of strain ATCC 25017 (GenBank ID: 27853, a project of the DOE Joint Genome Institute) and a draft genome of strain ATCC 25015^T^ (GenBank ID: 32411, a project of The Broad Institute) have been sequenced. Comparative genomic analyses of these strains with *F. novicida* and *F*. *tularensis* isolates have revealed major differences in metabolic competency and putative pathogenic characteristics [19,20]. In addition, a preliminary genomic sequence of *F. noatunensis* isolate GM2212 and its comparison to *F. philomiragia* have been reported [21]. The objectives of the present study were to sequence the genome of *Francisella* strain TX07-7308 and to identify the genetic differences between this strain and *F. philomiragia* strain ATCC 25017 using a comparative genomics approach. Since genetic relationships and evolutionary contexts could be better understood by whole-genome analyses of conserved operons, it was envisaged to include the genomes of different *Francisella* species and strains in the comparisons, when feasible.

## Methods

Bacterial cultivation and chromosomal DNA extraction were performed at the Centers for Disease Control and Prevention, Fort Collins, using standard procedures [13,22]. Genomic library construction, sequencing, and finishing were performed at the Genome Science Facilities of Los Alamos National Laboratory as described previously [23-25]. Prediction of the number of subsystems and pairwise BLAST comparisons of protein sets within strains TX07-7308 and ATCC 25017 were performed using the Rapid Annotation using Subsystems Technology (RAST), which is a fully automated, prokaryotic genome annotation service [26]. Proteins deemed to be specific to each strain were compared against the NCBI non-redundant protein database to determine whether they were hypothetical or conserved hypothetical. If there was no adequate alignment with any protein (less than 25% identity or aligned region is less than 25% of the predicted protein length), the translated open reading frame (ORF) was named a hypothetical protein.

Multiple genome comparisons were performed using the ‘progressive alignment’ option available in the program MAUVE version 2.3.0. Default scoring and parameters were used for generating the alignment. A synteny plot was generated using the program NUCmer. The program uses exact matching, clustering, and alignment extension strategies to create a dot plot based on the number of identical alignments between two genomes. Prophage regions (PRs) were identified using Prophinder (http://aclame.ulb.ac.be/Tools/Prophinder/), an algorithm that combines similarity searches, statistical detection of phage-gene enriched regions, and genomic context for prophage prediction. Insertion sequences (ISs) were identified by whole genome BLAST analysis of strains TX07-7308 and ATCC 25017 using the IS finder (http://www-is.biotoul.fr/). Gene acquisition and loss among the different strains were determined by comparing gene order, orientation of genes (forward/reverse), GC content of genes (% above or below whole genome average), features of intergenic regions (*e.g.*, remnants of IS elements, integration sites), and the similarity of proteins encoded by genes at a locus of interest (>90% identity at the predicted protein level for orthologs).

The Integrated Microbial Genomes System (http://img.jgi.doe.gov/cgi-bin/w/main.cgi) was used for comparing the genomes and identification of shared and unique protein encoding genes. The phylogenetic profiling tool within IMG was used to find single gene homologs. Three 3-way comparisons were performed using different genome orders, and the lowest number was used to represent the core genes in the venn diagram. Pairwise comparisons were performed bidirectionally for each pair of genomes and the lowest number was used in the diagram. Strain-specific genes were obtained by subtracting the larger number of common genes from the total number of genes in the genome. The specific parameters used for the analysis were: E-value: 1e-05; minimum percent identity: 30%; algorithm: by present/absent homologs; minimum taxon percent with homologs: 100%.

Multiple sequence alignments for phylogenetic analyses of strains TX07-7308 and ATCC 25017 were performed using the program MUSCLE available at the website http://www.phylogeny.fr/ [27,28]. The alignment was followed by a bootstrapped (n=100) neighbor joining method for inferring the phylogenies [29]. Only full-length 16S rRNA and succinate dehydrogenase (*sdhA*) gene sequences from high quality, finished *Francisella* genomes were included in these comparisons.

## Results and discussion

### General genome comparisons

**Figure 1:**
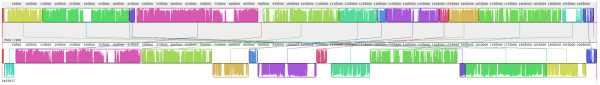
** Alignment of the chromosomes of strains TX07-7308 (top) and ATCC 25017 (bottom) using MAUVE 2.** Identically colored boxes, known as locally collinear blocks (LCBs), depict homologous regions in the two chromosomes. The edges of LCBs indicate chromosome rearrangements due to recombination, insertions, and/or inversions. Sequences of strain ATCC 25017 inverted in relation to those of strain TX07-7308 are shown as blocks below the horizontal line. The vertical lines connecting the LCBs point to regions of homology among the two chromosomes. Numbers above the maps indicate nucleotide positions within the respective chromosomes.

**Figure 2:**
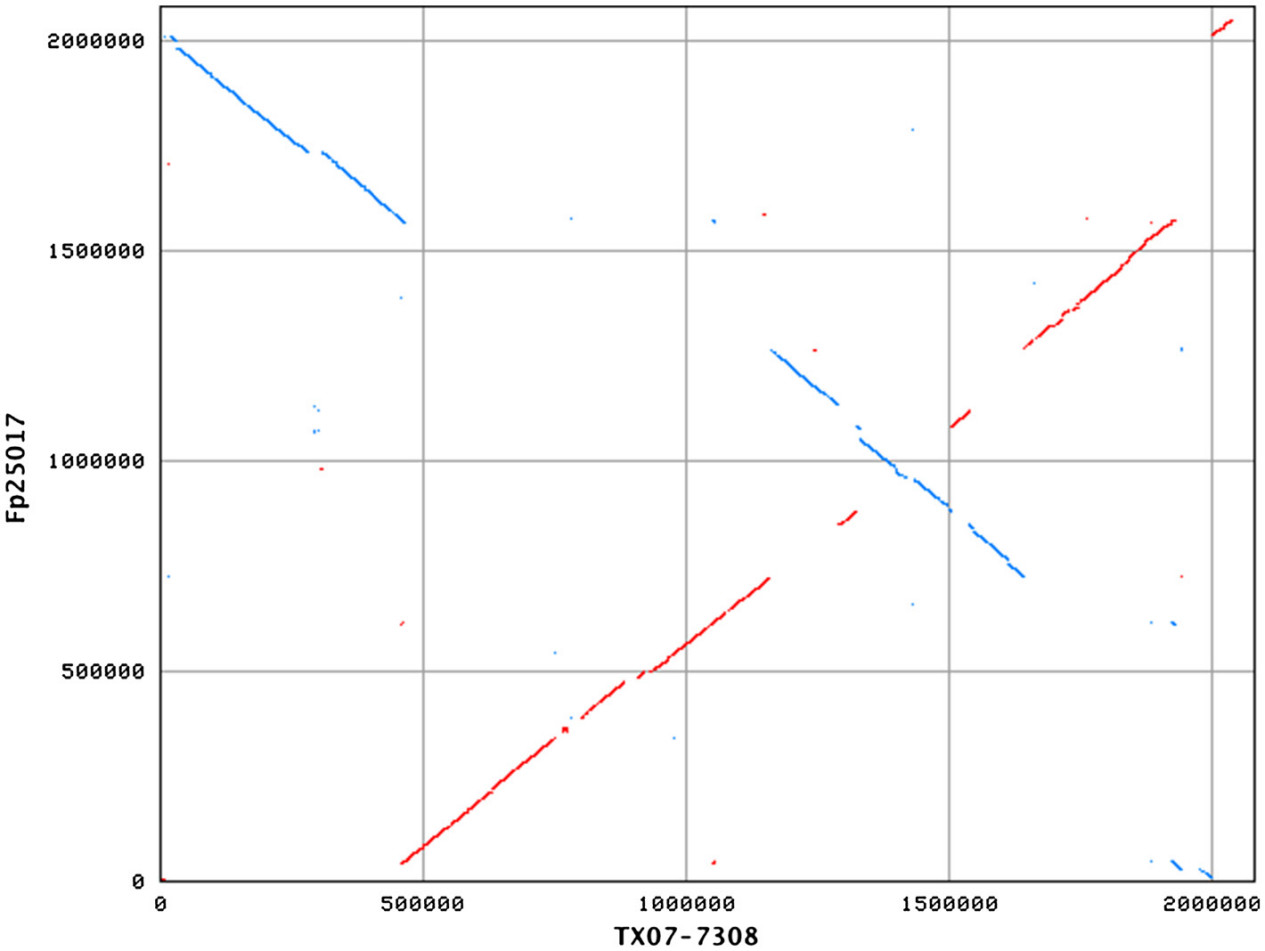
** Synteny plot of the chromosomes of strains TX07-7308 and ATCC 25017 generated by NUCmer.** This plot shows regions of identity between the two chromosomes based on pair-wise alignments. Strain TX07-7308 sequence is represented on the X-axis. Strain ATCC 25017 sequence is represented on the Y-axis. Plus strand matches are slanted from the bottom left to the upper right corner and are shown in red. Minus strand matches are slanted from the upper left to the lower right and are shown in blue. The number of dots/lines shown in the plot is the same as the number of exact matches found by NUCmer. Numbers indicate nucleotide positions within the respective chromosomes.

The chromosome of strain ATCC 25017 was 9,844 bp larger than that of strain TX07-7308. Although the chromosomes of strains ATCC 25017, ATCC 25015, and TX07-7308 had minor differences in size, their average GC content and the percentage of sequence that encodes proteins were similar (Table 1). Whole genome alignment using MAUVE showed the presence of extensive blocks of homologous regions among the chromosomes of strains ATCC 25017 and TX07-7308 (Figure 1). NUCmer analyses revealed that the chromosome of strain ATCC 25017 had many gaps, translocations, and/or inversions when compared to that of strain TX07-7308 (Figure 2). Detailed sequence examination indicated that some of these gaps and/or inversions were associated with integrative elements. Strain TX07-7308 contained a genomic island (920,244 bp to 927,148 bp, 29.89% GC) that was absent in strain ATCC 25017, but was present in *F. novicida* strain U112 (369,322 bp to 376,086 bp, 30.7% GC). Strain TX07-7308 contained yet another genomic island (1,940,355 bp to 1,971,897 bp, 28.98% GC) that was also absent in strain ATCC 25017, but was partially present in *F. novicida* strain U112 (*e.g.*, 40,574 bp to 61,538 bp, 28.8% GC). Strain ATCC 25017 contained a genomic island (1,981,253 bp to 1,996,171 bp, 30% GC) that was absent in strain TX07-7308 and *F. novicida* strain U112. Furthermore, strain TX07-7308 lacked plasmids, but strain ATCC 25017 contained a single plasmid [30]. 

**Table 1:** **Comparison of the genomes of five different strains within the genus *Francisella***

**Genome feature**	***F. philomiragia*ATCC 25017**	***Francisella*spp. TX07-7308**	***F. philomiragia*ATCC 25015**^**T**^	***F. novicida*U112**	***F*.*tularensis*WY96-3418**
Chromosome size (bp)	2045775	2035931	1985978	1910031	1898476
Number of protein-coding genes	1915	1976	1892	1733	1634
Overall coding density (%)	91	91	91	89	79
Number of subsystems^1^	306	302	308	253	250
GC content (%)	32.57	32.9	32.52	32.48	32.27
5S rRNA genes^2^	3+1	3+1	3+1	3+1	3+1
23S rRNA genes^2^	3	3	3	3	3
16S rRNA genes^2^	3	3	3	3	3
tRNA genes	39	39	35	38	38
GenBank accession number	CP000937	CP002872	ABYY00000000	CP000439	CP000608

^1.^Type strain, draft genome.

^1^ Subsystems predicted by RAST server.

^2^ Each strain contains three rRNA operons (5S-23S-16S) and an orphan 5S rRNA fragment.

**Figure 3:**
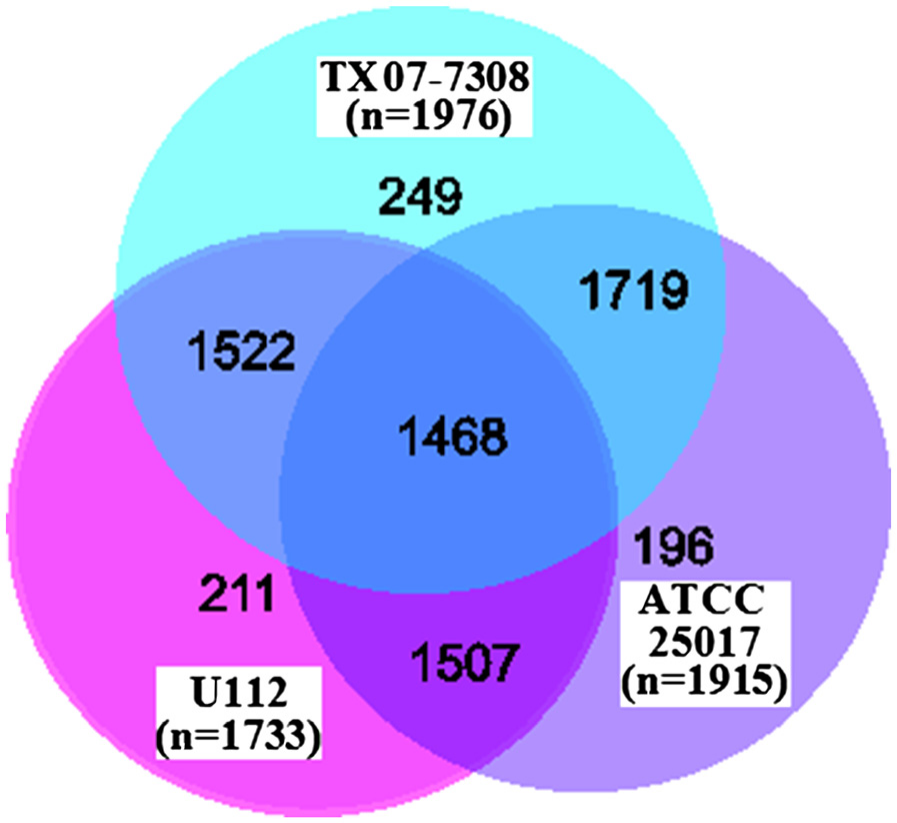
** Venn diagram depicting the shared (orthologs) and unique protein coding genes in the genomes of *Francisella* spp. strain TX07-7308, *F. philomiragia* strains ATCC 25017, and *F. novicida* strain U112.** Numbers in parenthesis indicate the total number of protein coding genes in the genomes of each of the three strains. Strains ATCC 25017 and TX07-7308 had 1719 orthologous protein coding genes, strains ATCC 25017 and U112 had 1507 orthologous protein coding genes, whereas strains TX07-7308 and U112 had 1522 orthologous protein coding genes.

A three-way comparison of the chromosomes of strains U112, ATCC 25017, and TX07-7308 revealed that they encoded 1,468 orthologous protein-coding genes (bidirectional best hits). Strain ATCC 25017 chromosome encoded 196 protein-coding genes with no homologs in strains TX07-7308 and U112. Strain TX07-7308 chromosome encoded 249 protein-coding genes with no homologs in strains U112 and ATCC 25017. Strain U112 encoded 211 protein-coding genes with no homologs in strains TX07-7308 and ATCC 25017 (Figure 3). In strain ATCC 25017, 507 predicted genes could not be assigned a function based on BLAST analysis and have therefore been annotated as encoding hypothetical or conserved hypothetical proteins. In strain TX07-7308, 447 predicted genes have been annotated as encoding hypothetical or conserved hypothetical proteins.

### Ribosomal RNA operons and tRNA genes

Bacterial rRNA operons generally contain the 16S, 23S, and 5S rRNA genes linked together by one or more tRNA genes located in the internal transcribed spacer [31]. Chromosomes of most members of the genus *Francisella* sequenced to date, including strains ATCC 25017 and TX07-7308, contained three copies of the 16S rRNA-tRNA^Ile^-tRNA^Ala^-23S rRNA-5S rRNA operon. Although the three rRNA operon sequences were remarkably homogenous within any genome, some degree of heterogeneity was obvious between the *F. philomiragia* and *F. tularensis/novicida* genomes (data not shown). Notably, the chromosomes of strains ATCC 25017 and TX07-7308 contained an unlinked 119 bp 5S rRNA sequence (380,981 bp to 381,099 bp and 1,883,624 bp to 1,883,742 bp, respectively) that was also present in other members of the genus *Francisella* (*e.g.*, FTN_0501 in *F. novicida* strain U112 and FTT_r01 in *F. tularensis* strain Schu S4). Furthermore, the positions of the three rRNA operons were not conserved in the chromosomes of strains ATCC 25017 and TX07-7308, but they were relatively conserved among the chromosomes of *F. novicida* and *F. tularensis* strains.

A positive correlation between the abundance of tRNA and rRNA genes among bacterial genomes has been observed [32]. Typically, bacteria with three 16S rRNA genes are expected to contain ~40 tRNA genes [31]. Not surprisingly, members of the genus *Francisella* contain 37–39 tRNA genes (~1.8% of the genome), representing all 20 amino acids. Although some redundancy of tRNA genes was apparent among all *Francisella* genomes (*e.g.*, four copies of tRNA^Leu^), they each contained a single copy of tRNA^Asn^, tRNA^Asp^, tRNA^Cys^, tRNA^Gln^, tRNA^Glu^, tRNA^His^, tRNA^Phe^, tRNA^Pro^, tRNA^Trp^, and tRNA^Tyr^ genes. In all of the publicly available *Francisella* genomes, genes encoding tRNA^Ile^ and tRNA^Ala^ were never present outside of the rRNA operons. This is generally true of most other bacteria and the occurrence of genes encoding tRNA^Ile^ and tRNA^Ala^ genes within the rRNA operons has also been noted in *Escherichia coli* K12 and *Rhodopseudomonas palustris*[33,34]. Furthermore, almost all *Francisella* genomes contained genes encoding putative aminoacyl tRNA synthetases specific for each of the 20 amino acids. They also contained a single gene encoding a putative peptidyl-tRNA hydrolase (*e.g.*, Fphi_1584, FTN_1003, and FTT_0680c).

### Integrative and transposable elements

Short sequence repeats, which include insertion sequences (IS), are the hallmark of *F. tularensis* genomes and these IS elements are thought to be generally stable among different isolates despite their diverse geographical origins [17,35]. Pair-wise BLASTN analyses indicated that IS elements were less abundant in the genome of strain TX07-7308 in comparison to those of *F. tularensis* strains Schu S4 and OSU18, which contained at least six categories of *IS* elements (*IS*Ftu1-*IS*Ftu6). Whole genome BLASTP analyses showed that strains ATCC 25017 and TX07-7308 each contained a single copy of the *IS*Ftu2 sequence (Fphi_0257, 247 aa; F7308_1638, 235 aa; 91% identity), but they lacked *IS*Ftu1 and *IS*Ftu3-*IS*Ftu6 sequences ( Additional file 1: Table S1). Furthermore, strains TX07-7308 and ATCC 25017 contained at least 4 truncated loci related to *IS*1016 (F7308_1212, F7308_1886, F7308_1888, and Fphi_1491; Additional file 1: Table S1).

In strain TX07-7308, the locus adjacent to F7308_1638 encoded an integral membrane protein (F7308_1634, 343 aa), a phosphoserine phosphatase (F7308_1635, 216 aa), a lignostilbene-alpha, beta-dioxygenase (F7308_1636, 99 aa), and a beta carotene dioxygenase (F7308_1637, 344 aa). Interestingly, the homologs of these ORFs in strain ATCC 25017 (Fphi_0135-0138) were associated with Fphi_0139, which encoded a putative integrase (314 aa). Strain ATCC 25017 contained 41 full-length and 5 truncated orthologs of Fphi_0139 ( Additional file 1: Table S1). Whereas an ortholog of Fphi_0139 was present in *Francisella piscicida* strain GM2212 (GenBank locus EU492905, protein_id ACA58079), it was absent in all other publicly available *Francisella* genomes, including strain ATCC 25015.

Strains ATCC 25017 and TX07-7308 also contained a putative mutator family transposase (Fphi_0511, 410 aa; F7308_0014, 211 aa; 85% identity, Additional file 1: Table S1) that was absent in all other publicly available *Francisella* genomes, except strain ATCC 25015. In strain ATCC 25017, this transposable element was associated with a gene encoding a putative chitinase (Fphi_0512, 617 aa) whose ortholog was present in strain ATCC 25015, but not in strain TX07-7308 and all other publicly available *Francisella* genomes. Strain TX07-7308 mutator family transposase was associated with genes encoding a putative chitinase (F7308_0017, 942 aa) and a two-component response regulator (F7308_0016, 239 aa) whose orthologs were present in strain ATCC 25015, but not in strain ATCC 25017 and all other publicly available *Francisella* genomes. Furthermore, strain ATCC 25017 contained a locus ( Additional file 1: Table S1) encoding a putative Tn*1546* family transposase (Fphi_1740, 552 aa), an integrase (Fphi_1741, ortholog of Fphi_0139, 314 aa), a Tn*3* family transposase (Fphi_1742, 238 aa), a transposase-like protein (Fphi_1743, 137 aa), and an invertase/recombinase/resolvase-like protein (Fphi_1744, 189 aa). These genes were absent in all other publicly available complete *Francisella* genomes, including strain TX07-7308. Several genes encoding putative phage integrases were also identified in the genomes of strains ATCC 25017 and TX07-7308 ( Additional file 1: Table S1).

### Type IV pili, oligopeptide ABC transporter system, *Francisella* pathogenicity island, and iron metabolism genes

Several species of pathogenic gram-negative bacteria contain type IV pili, also known as fimbriae, which facilitate bacterial adhesion to host cells, biofilm formation, and twitching motility [36]. Structures resembling type IV pili have been observed on the surface of *F. tularensis* live vaccine strain (LVS) and *F. novicida* strain U112 using transmission electron microscopy [37,38]. Recent evidence suggests that type IV pili are required for virulence of *F. tularensis* and comparisons of available *Francisella* genomes have revealed the heterogeneity of genes putatively involved in the assembly of type IV pili [39,40]. Strains ATCC 25017 and TX07-7308 contained several ORFs that encoded putative proteins related to the assembly of type IV pili (Table 2). Although most of these genes were found in different locations on the two chromosomes, two gene clusters were conspicuous from gross comparisons (Fphi_0006-0010/F7308_1236-1232 and Fphi_0422-0424/F7308_0429-0427). Since many of the genes putatively involved in the biosynthesis of type IV pili in strains ATCC 25017 and TX07-7308 were also present in *F. novicida* and *F. tularensis* strains (data not shown), the biochemical and functional similarity of type IV pili among these bacteria cannot be ruled out. Nevertheless, the role of type IV pili in the survival of *F. philomiragia* in the environment needs to be carefully studied, especially in light of recent reports of the ability of this bacterium to form biofilms and interact with the aquatic protist *Acanthamoeba castellanii*[41]. 

**Table 2:** Identification of pilus biosynthesis, oligopeptide transport, glucuronate metabolism, and lactose metabolism genes

**Function** Locus tag (Protein)	**Annotation**	**Closest homolog outside*Francisella locus tag, protein, identity, E-value***
**Pilus biosynthesis**^1^		
Fphi_0006 (582 aa)	Type IV pili secretin component (PilQ)	Csal_0612, 668 aa, 36%, 7e-84
Fphi_0007 (196 aa)	Type IV pili lipoprotein (PilP)	Rmet_3269, 179 aa, 37%, 2e-05
Fphi_0008 (199 aa)	Type IV pili glycosylation protein (PilO)	Lferr_0885, 217 aa, 24%, 2e-04
Fphi_0009 (187 aa)	Type IV pili associated protein (PilN)	Lferr_0888, 197 aa, 26%, 5e-05
Fphi_0010 (334 aa)	Type IV pili, pilus assembly protein	None
Fphi_0117 (592 aa)	Type IV pili ATPase (PilB/PulE)	Shewmr4_0420, 569 aa, 48%, 4e-147
Fphi_0118 (410 aa)	Type IV pili polytopic inner membrane protein (PilC/PulF)	Tgr7_0786, 403 aa, 42%, 7e-85
Fphi_0157 (194 aa)	Type IV pili, pilus assembly protein (FimT)	Shal_1142, 171 aa, 30%, 6e-11
Fphi_0422 (152 aa)	Type IV pili fiber building block protein (PilA/PilE)	Noc_2540, 147 aa, 35%, 7e-15
Fphi_0423 (145 aa)	Type IV pili, pilus assembly protein (PilA/PilE)	CV_4209, 155 aa, 33%, 5e-12
Fphi_0424 (409 aa)	Type IV pili, pilus assembly protein (PilA/PilE)	NMB0018, 170 aa, 30%, 3e-09
Fphi_0449 (313 aa)	Type IV pili, pilus assembly protein (PilA/PilE)	CtCNB1_4760, 144 aa, 28%, 3e-05
Fphi_0522 (304 aa)	Type IV pilus assembly protein PilW	None
Fphi_0763 (149 aa)	Type IV pili, pilus assembly protein	Tola_2518, 128 aa, 36%, 2e-15
Fphi_0996 (342 aa)	Twitching motility protein (PilT)	SO_3351, 345 aa, 58%, 4e-106
Fphi_1136 (337 aa)	Type IV pilus assembly protein (PilW)	None
Fphi_1587 (282 aa)	Type IV pili leader peptidase and methylase (PilD)	Maqu_2683, 291 aa, 41%, 9e-55
Fphi_1689 (297 aa)	Type IV pilus biogenesis/stability protein (PilW)	Kkor_1840, 263 aa, 30%, 1e-09
Fphi_1748 (111 aa)	Type IV pilin (PilA)	Hsero_0660, 168 aa, 28%, 3e-06
**Oligopeptide transport**^2^		
Fphi_1024 (558 aa)	ABC-type oligopeptide transport system, periplasmic component (OppA)	Rahaq_2640, 546 aa, 38%, 1e-90
Fphi_1025 (312 aa)	Oligopeptide ABC transporter inner membrane protein (OppB)	EAMY_1936, 306 aa, 47%, 3e-77
Fphi_1026 (286 aa)	Oligopeptide transport system permease protein (OppC)	VP2089, 300 aa, 53%, 2e-84
Fphi_1027 (219 aa)	Oligopeptide transport system permease protein (OppD)	CV_4326, 333 aa, 61%, 3e-137
Fphi_1029 (324 aa)	Oligopeptide transport ATP-binding protein (OppF)	NT05HA_1387, 332 aa, 58%, 5e-105
**Glucuronate metabolism**^3^		
F7308_1388 (520 aa)	Rhamnogalacturonide transporter (RhiT)	KPK_1307, 502 aa, 44%, 2e-116
F7308_1389 (776 aa)	Alpha-glucosidase	BL00280, 802 aa, 50%, 0.0
F7308_1390 (490 aa)	D-mannonate oxidoreductase (UxuB)	CJA_0180, 492 aa, 42%, 3e-105
F7308_1391 (396 aa)	Mannonate dehydratase (UxuA)	PROSTU_04181, 396 aa, 59%, 5e-135
F7308_1392 (321 aa)	2-keto-3-deoxygluconate kinase (KdgK)	Sde_1269, 296 aa, 43%, 9e-59
F7308_1393 (182 aa)	KDPG Aldolase (KdgA)	BC1003_2949, 213 aa, 37%, 4e-34
F7308_1394 (471 aa)	Glucuronate isomerase (UxaC)	Sde_1272, 471 aa, 51%, 3e-140
F7308_1395 (463 aa)	D-xylose-proton symporter (XylT)	CBUD_1731, 463 aa, 43%, 3e-91
F7308_1396 (325 aa)	Inositol oxygenase	56727 Miox, 285 aa, 37%, 3e-47
**Lactose metabolism**^4^		
Fphi_0309 (655 aa)	Beta-galactosidase	BMD_1886, 651 aa, 42%, 2e-159
Fphi_0310 (394 aa)	Sugar transport protein	ZP_07631419, 394 aa, 31%, 2e-44

^1^Homologs of these genes are also found in strains TX07-7308 and ATCC 25015.

^2^Homologs of these genes are also found in strain ATCC 25015, but absent in strain TX07-7308.

^3^Homologs of these genes are also found in strain ATCC 25015, but absent in strain ATCC 25017.

^4^Homologs of these genes are also found in strain ATCC 25015, but absent in strain TX07-7308.

Several bacterial species possess oligopeptide permeases (Opp), which are ATP-binding cassette transporters of oligopeptides [42]. The imported oligopeptides serve as a source of nitrogen for the cell and may also have a role in signal transduction and pathogenesis [43]. A typical bacterial oligopeptide transport system consists of five proteins encoded by the *oppABCDF* operon [42,43]. It has been reported that *oppD* and *oppF* occur as separate ORFs in Type A strains of *F. tularensis*, but a 960 bp internal deletion has caused the disruption of *oppD* and *oppF* coding sequences in Type B strains of *F. tularensis*[19]. Genome comparisons indicated that *F. philomiragia* strain ATCC 25017 and *F. novicida* strain U112 contained an *opp* locus (Fphi_1024-1029 and FTN_1589-1593, respectively). Within this locus, FTN_1590 encoded a putative full-length OppD protein (322 aa), but Fphi_1027 was annotated as a pseudogene because of a frame-shift mutation (Table 2). Although the *opp* locus was present in a complete or truncated form in most currently available *Francisella* genomes, it was absent in strain TX07-7308. It is possible that strain TX07-7308 lost the ancestral *opp* locus during reductive evolution or it represents a lineage that evolved before the acquisition of the *opp* locus by the *F. philomiragia* clade.

A cluster of 17–19 genes has been proposed to constitute the *Francisella* pathogenicity island (FPI) and is found in a single copy in *F. novicida* strain U112 (FTN_1309-1325), but is duplicated in the genomes of *F. tularensis*[17,44]. Genome comparisons revealed that strains ATCC 25017 and TX07-7308 also contained a cluster of genes related to the FPI (Fphi_1363-1367/F7308_1001-1005 and Fphi_1369-1377/F7308_1007-1016). The predicted proteins within the putative FPIs of strains ATCC 25017 and TX07-7308 had an average identity of 84.5% (the identity range was 59-96%). The order and orientation of genes within the FPI of strains ATCC 25017 and TX07-7308 were similar. Furthermore, the homolog of FTN_1314, which encodes a hypothetical protein in *F. novicida* strain U112, was truncated in strain TX07-7308 (F7308_1011) and homologs of FTN_1318-FTN_1320 (encoding a hypothetical protein, a pathogenicity determinant protein, and a hypothetical protein, respectively) were absent in strains ATCC 25017 and TX07-7308.

The biosynthesis of a polycarboxylate siderophore in *F. tularensis* strain Schu S4 and *F. novicida* strain U112 has been described previously [45,46]. Strains ATCC 25017 and TX07-7308 contained the ferric uptake regulator gene (*fur*, Fphi_0928/F7308_0528) and a cluster of genes putatively involved in the biosynthesis of a polycarboxylate siderophore (Fphi_0922-0926 and F7308_0522-0526). An ortholog of strain Schu S4 *fupA* (FTT_0918), whose product is required for efficient utilization of siderophore-bound iron [47], was also found in strains ATCC 25017 and TX07-7308 (Fphi_0393/F7308_0459). The presence of FPI-related genes, *fur*, and *fupA* in almost all *Francisella* genomes suggests that these functions are essential for their survival in the environment and/or host.

### Genetics of uronic acid metabolism

Some bacteria have evolved mechanisms for the metabolism of uronic acids and uronates using the Entner-Doudoroff pathway [48]. In this pathway, α-D-glucuronic acid (GlcUA) is converted into 2-keto-3-deoxygluconate (KDG) by a three-step process. The subsequent phosphorylation of KDG yields 2-keto-3-deoxy-6-phosphogluconate (KDPG), which is finally cleaved to produce glyceraldehyde 3-phosphate (G3P) and pyruvate. Comparative genomic analyses indicated that strains TX07-7308 and ATCC 25015 contained a cluster of nine ORFs that appeared to constitute a polycistronic operon (F7308_1388-1396). These ORFs encoded putative proteins related to enzymes involved in GlcUA catabolism (Table 2). Similar operons were also identified in *F. novicida*-like strains Fx1 and 3523 [18].

The predicted mannonate dehydratase, 2-keto-3-deoxygluconokinase, KDPG aldolase, and glucuronate isomerase proteins from strain TX07-7308 had 57%, 37%, 36%, and 28% identities to *E. coli* UxuA (ECs5281), KdgK (ECs4406), KdgA (ECs2560), and UxaC (ECs3974), respectively (E-values = 8e-136 to 3e-29). These enzymes catalyze the dehydration of D-mannonate to KDG, phosphorylation of KDG, cleavage of KDPG to pyruvate and G3P, and the conversion of D-glucuronate to D-fructuronate, respectively [48]. The GlcUA utilization gene cluster of strain TX07-7308 had some similarities to that of *Bacillus stearothermophilus* T-6, which has been predicted to metabolize GlcUA akin to *E. coli* and *Bacillus sutbtilis*[49].

The ORFs encoding a putative inositol oxygenase in strains TX07-7308 and ATCC 25015 had no bacterial homologs in the public databases outside of the genus *Francisella*. However, strain TX07-7308 inositol oxygenase had 37% identity to *Mus musculus myo*-inositol oxygenase (56727 Miox, E-value = 5e-54), which catalyzes the conversion of *myo*-inositol to GlcUA [50]. *Myo*-inositol and its derivatives are ubiquitous among eukaryotes and archaea, but their synthesis and metabolism is believed to be less common among bacteria [51]. Although none of the *F. tularensis* genomes sequenced to date had ORFs encoding proteins putatively involved in the transport and/or metabolism of *myo*-inositol, most of them had a *suhB* homolog (*e.g.*, FTT_1382 in strain Schu S4). A *suhB* homolog was also found in the genomes of strains TX07-7308 and ATCC 25017 (F7308_0980 and Fphi_1342). This evolutionarily conserved gene encoded inositol-1-monophosphatase, which hydrolyzes *myo*-inositol-1-phosphate to yield free *myo*-inositol [52,53]. Thus, it appears that most members of *Francisella* can convert *myo*-inositol-1-phosphate to free *myo*-inositol. However, only some strains may be able to utilize *myo*-inositol to synthesize GlcUA, which is then metabolized using the Entner-Doudoroff pathway.

### Atypical *lac* operons

The *lac* operon, which contains the structural genes encoding proteins that facilitate lactose metabolism, is found in a variety of bacteria. Lactose is imported into the cell as a free sugar by means of a permease and the enzyme β-galactosidase hydrolyzes this disaccharide into galactose and glucose [54]. Genome comparisons revealed that strains ATCC 25015 and ATCC 25017, but not strain TX07-7308, contained a cluster of two ORFs that appeared to constitute an operon (Fphi_0309-0310, Table 2). Similar *lac* operons were also identified in *F. novicida*-like strains Fx1 and 3523 [18]. Strain ATCC 25017 predicted LacZ had 29% identity to the β-galactosidase (AAF16519, BgaB, E-value = 2e-79) of *Carnobacterium maltaromaticum*[55] and 26% identity to the β-galactosidase (O07012.2, GanA, E-value = 8e-76) of *Bacillus subtilis*[56], but was unrelated to the β-galactosidase of *E. coli*. Strain ATCC 25017 LacY had 25% identity to the putative oligogalacturonide transporter (NP_752266, E-value = 3e-07) of *E. coli* CFT073 and 22% identity to the putative sugar transporter (YP_081973, E-value = 1e-08) of *Bacillus cereus*. However, genes encoding the galactoside O-acetyltransferase (*lacA*) and the regulatory protein (*lacI*) were not found in strains ATCC 25015 and ATCC 25017. In strain ATCC 25017, an ORF (Fphi_0306) encoding a putative transposase/integrase-like protein was found near the *lac* operon. Furthermore, several *F. tularensis* genomes (*e.g.*, strains Schu S4, WY96-3418, OSU18, and FSC147) contained a truncated/vestigial *lacZ*, but lacked a *lacY* (data not shown).

Although the classic *lac* operon of *E. coli* consists of three ORFs (*lacZYA*), which are regulated by the product of the adjacent *lacI* repressor gene, bacteria containing only *lacZY* or *lacZ* have been identified [57-59]. While the evolutionary origin of the *E. coli lac* operon is uncertain [59], the occurrence of *lac* genes near integrative and conjugative elements and the identification of *E. coli*-like *lac* operons in some gram-positive bacteria suggest their lateral mobility [60-62]. From genome comparisons, it appeared that the last common lactose-utilizing ancestor of *F. philomiragia* strain ATCC 25017 and *F. tularensis* strains may have acquired the *lacZY* operon by transposon-mediated horizontal transfer. Nevertheless, detailed phylogenetic analyses are required to establish the evolutionary origin of the *Francisella lac* operon. The loss of the *lacZY* operon in most *F. tularensis* strains may be due to niche selection or through genetic drift, and a similar mechanism for some members of *Enterobacteriaceae* has been proposed [59]. The ability to metabolize lactose probably affords strains ATCC 25015 and ATCC 25017 a growth advantage in environments where the sugar is present and these may represent a subset of *Francisella* that have retained an ancestral copy of the *lac* operon. The *lacZ* gene identified in this report could be useful in studies involving pathogenic *F. tularensis* strains that require a native reporter protein.

### Genetics of thiamine, riboflavin, folate, biotin, and siroheme biosynthesis

Thiamine pyrophosphate (Vitamin B_1_) is involved in several microbial metabolic functions [63]. Prokaryotes have evolved elaborate mechanisms to either synthesize this important co-factor *de novo* or acquire it from their niche [64]. Thiamine biosynthesis (TBS) in most bacteria is accomplished by two major pathways; one involves the formation of hydroxymethylpyrimidine pyrophospate (HMP-PP) from aminoimidazole ribotide using ThiC and ThiD and the other involves the formation of hydroxyethylthiazole phosphate (HET-P) using ThiS, ThiF, ThiG, and ThiO. The enzyme thiamine phosphate synthase (ThiE) combines HMP-PP and HET-P to produce thiamine phosphate, which is phosphorylated by thiamine monophosphate kinase (ThiL) to produce thiamine pyrophosphate [64].

Strains ATCC 25017 and TX07-7308 contained an operon with six ORFs (*thiCOSGDF*, Fphi_0086-0090 and F7308_1155-1160, respectively) encoding proteins related to enzymes involved in thiamine biosynthesis in several prokaryotes (Table 3). A similar *thiCOSGDF* operon was also identified in *F. novicida*-like strain 3523 [18]. The genetic organization of strains ATCC 25017 and TX07-7308 *thiCOSGDF* locus was similar to the plasmid-encoded *thiCOGE* locus involved in thiamine biosynthesis in *Rhizobium etli*[65]. An analogous gene cluster (*thiOGF)* is found within plasmid pEA29 of the plant pathogen *Erwinia amylovora* strain Ea88 [66]. The chromosome of the lithoautotrophic bacterium *Ralstonia eutropha* H16 also contains a *thiCOSGE* locus that is proposed to be involved in *de novo* synthesis of thiamine [67]. At the protein level, strain ATCC 25017 ThiC was 68% identical to ThiC of *R. etli* (AAC45972) and *Ra. eutropha* (H16_A0235), whereas strain ATCC 25017 ThiF was 34% identical to ThiF of *E*. *amylovora* (NP_981993, E-value = 4e-17). Furthermore, ATCC 25017 ThiO and ThiS were 30% identical to ThiO (H16_A0236) and ThiS (H16_A0237) of *Ra. eutropha*, respectively (E-values = 4e-34 to 2e-10). Putative thiazole synthase ThiG of strain ATCC 25017 was ~51% identical to ThiG of *R. etli* (AAC45974, E-value = 1e-70) and *Ra. eutropha* (H16_A0238, E-value = 1e-81). 

**Table 3:** Identification of genes putatively involved in the biosynthesis of various vitamins and siroheme

**Function** Locus tag (Protein)	**Annotation**	**Closest homolog outside*Francisella locus tag, protein, identity,E-value***
**Thiamine biosynthesis**		
		
Fphi_0086 (251 aa)	Thiazole biosynthesis adenylyltransferase (ThiF)	Mrub_1727, 266 aa, 38%, 7e-41
Fphi_0087 (485 aa)	Fused protein Phosphomethylpyrimidine kinase (ThiD)/Thiamine-phosphate pyrophosphorylase (ThiE)	lpg1568, 495 aa, 35%, 1e-72
Fphi_0088 (259 aa)	Thiazole synthase (ThiG)	lpg1567, 263 aa, 58%, 2e-86
Fphi_1923 (66 aa)	Thiamine biosynthesis protein (ThiS)	IL0768, 66 aa, 32%, 1e-04
Fphi_0089 (350 aa)	Thiamine biosynthesis oxidoreductase (ThiO)	Kkor_0127, 351 aa, 34%, 3e-47
Fphi_0090 (592 aa)	Thiamine biosynthesis protein (ThiC)	CV_0235, 632 aa, 72%, 0.0
**Riboflavin biosynthesis**		
Fphi_0395 (306 aa)	Riboflavin kinase (RibF)	D11S_1924, 308 aa, 47%, 5e-69
Fphi_0713 (356 aa)	Riboflavin biosynthesis protein (RibD)	Acear_1431, 371 aa, 44%, 5e-79
Fphi_0714 (201 aa)	Riboflavin synthase, alpha subunit (RibC/RibE)	CLD_1677, 228 aa, 48%, 5e-41
Fphi_0715 (403 aa)	GTP cyclohydrolase II (RibA)	DEFDS_1098, 405 aa, 49%, 5e-104
Fphi_0716 (147 aa)	6,7-dimethyl-8-ribityllumazine synthase (RibH)	lpg1180, 155 aa, 56%, 3e-39
**Folate biosynthesis**		
Fphi_0420 (282 aa)	Methenyltetrahydrofolate cyclohydrolase (FolD)	SULAZ_0428, 284 aa, 60%, 3e-87
Fphi_0547 (394 aa)	Dihydrofolate synthase (FolC)	Ssed_1653, 421 aa, 34%, 2e-45
Fphi_0600 (165 aa)	Dihydrofolate reductase (FolA)	BMD_4044, 161 aa, 54%, 7e-41
Fphi_1791 (184 aa)	Para-aminobenzoate synthase, amidotransferase component	Slin_0655, 205 aa, 38%, 4e-37
Fphi_1792 (587 aa)	Para-aminobenzoate synthase, aminase component	Csal_2692, 629 aa, 37%, 3e-99
Fphi_1794 (117 aa)	Dihydroneopterin aldolase (FolB)	WPa_0696, 125 aa, 32%, 1e-09
Fphi_1795 (421 aa)	Dihydropteroate synthase (FolKP)	RBE_0032, 438 aa, 35%, 4e-48
**Biotin biosynthesis**		
Fphi_1798 (428 aa)	Adenosylmethionine-8-amino-7-oxononanoate aminotransferase (BioA)	CBU_1008, 442 aa, 51%, 4e-121
Fphi_1799 (313 aa)	Biotin synthase (BioB)	CBU_1007, 321 aa, 61%, 7e-103
Fphi_1800 (372 aa)	8-amino-7-oxononanoate synthase (BioF)	Sde_3138, 397 aa, 38%, 6e-60
Fphi_1801 (244 aa)	Biotin biosynthesis protein (BioC)	CBU_1004, 248 aa, 28%, 2e-10
Fphi_1803 (226 aa)	Dethiobiotin synthase (BioD)	Sde_3135, 230 aa, 38%, 5e-37
**Siroheme biosynthesis**		
Fphi_0284 (323 aa)	Porphobilinogen synthase (HemB)	Amet_0062, 324 aa, 60%, 1e-110
Fphi_0603 (469 aa)	Glutamyl-tRNA synthetase (GltX)	Fbal_2571, 470 aa, 60%, 2e-163
Fphi_0691 (300 aa)	Porphobilinogen deaminase (HemC)	APL_1010, 309 aa, 56%, 4e-86
Fphi_0945 (344 aa)	Uroporphyrinogen decarboxylase (HemE)	Rmag_1026, 347 aa, 50%, 1e-97
Fphi_1071 (414 aa)	Glutamyl-tRNA reductase (HemA)	PSPA7_5315, 422 aa, 35%, 2e-70
Fphi_1313 (252 aa)	Uroporphyrinogen-III synthase (HemD)	PSM_A0104, 613 aa, 31%, 1e-08
Fphi_1400 (400 aa)	Protoporphyrinogen oxidase (HemY/HemG)	Cphamn1_2137, 396 aa, 34%, 2e-58
Fphi_1812 (433 aa)	Glutamate-1-semialdehyde aminotransferase (HemL)	APJL_1583, 426 aa, 61%, 2e-151
Fphi_1842 (308 aa)	Coproporphyrinogen III oxidase (HemF)	Tcr_0017, 323 aa, 59%, 2e-106
Fphi_1900 (338 aa)	Ferrochelatase, protoheme ferro-lyase (HemH)	SMc04019, 342 aa, 50%, 7e-92

In strains ATCC 25017 and TX07-7308, Fphi_0087 and F7308_1159, respectively, appeared to encode a putative fused protein containing hydroxy-phosphomethylpyrimidine kinase and thiamine-phosphate pyrophosphorylase domains. In some bacteria, these functions are encoded by two different ORFs (*thiD* and *thiE*, respectively). Homologs of Fphi_0087 or F7308_1159 were found in several bacteria (*e.g.*, *Legionella pneumophila*, *Coxiella burnetii*, *Geobacter sulfurreducens*, and *Colwellia psychrerythraea*, ~30% protein identity) and plants (*e.g.*, *Arabidopsis thaliana*, *Zea mays*, and *Brassica napus*, ~29% protein identity). It has been proposed that these bifunctional enzymes are involved in the synthesis of HMP-PP as well as the condensation of HMP-PP and HET-P to produce thiamine monophosphate [63,68,69]. The 5^′^ untranslated regions of operons involved in thiamine biosynthesis and transport have been shown to contain a regulatory element called THI-box sequence [70]. Based on alignment of conserved sequences upstream of operons involved in thiamine biosynthesis from various bacteria, a putative THI-box sequence (5’-ACCCTTTGAACCTGATCTAGTTAGCACTAGTGTAGG-3’) was identified upstream of *thiC* in strains TX07-7308 and ATCC 25017. This suggests a thiamine-dependent regulation of *thiC* in strains ATCC 25017 and TX07-7308, as in other bacteria that have THI-box sequences upstream of TBS genes [63].

In bacteria that lack a TBS pathway, thiamine kinases may facilitate the salvage of dephosphorylated thiamine intermediates from the environment or growth medium [71]. A gene that encoded a putative thiamine pyrophosphokinase (TPK) was found in most members of the genus *Francisella*, including strains ATCC 25017 and TX07-7308 (Fphi_0159 and F7308_1656, respectively). Strain ATCC 25017 TPK was 27% identical to *Bacillus subtilis* TPK (THIN_BACSU, E-value = 1e-10), which catalyzes the direct conversion of thiamine to thiamine pyrophosphate [72]. Thus it appeared that most members of the genus *Francisella* are capable of salvage of dephosphorylated thiamine intermediates, but some strains (*e.g.*, ATCC 25017 and TX07-7308) can synthesize thiamine *de novo*, when thiamine intermediates are not available in their environments.

Vitamin B_2_ (riboflavin) is the precursor of coenzymes flavin mononucleotide (FMN) and flavin adenine dinucleotide (FAD), which are cofactors for several biochemical reactions [73]. Most bacteria, fungi, and plants can synthesize riboflavin *de novo* using one molecule of GTP and two molecules of ribulose 5-phosphate as substrates [74]. Strains ATCC 25017 and TX07-7308 contained five genes encoding enzymes putatively involved in riboflavin biosynthesis, of which four (Fphi_0713-0716) were within a single locus (Table 3). Tetrahydrofolate participates in a number of biochemical reactions and reduced folate cofactors are required for the biosynthesis of a variety of molecules in both prokaryotes and eukaryotes [75,76]. The production of folate involves several enzymes catalyzing the pterin and para-aminobenzoic acid branches of the pathway [77]. Strains ATCC 25017 and TX07-7308 contained seven genes encoding enzymes putatively involved in folate biosynthesis, of which four (Fphi_1791-1795) were clustered together (Table 3).

Vitamin H, commonly known as biotin, acts as a coenzyme in several enzyme-catalyzed carboxylation and decarboxylation reactions [78]. Whereas most bacteria can synthesize biotin *de novo* using pimelic acid as a precursor, some others have evolved mechanisms for importing this essential cofactor from their natural environments [79,80]. In the classic *bioABFCD* operon of *E. coli*, the *bioA* and *bioBFCD* genes are divergently transcribed and encode the enzymes catalyzing the biosynthesis of biotin [81,82]. The chromosomes of strains ATCC 25017 and TX07-7308 contained a cluster of genes that resembled the *bioABFCD* operon of *E. coli* (Fphi_1798-1803 and F7308_1318- 1314, respectively, Table 3). The *bioABFCD* operons of strains ATCC 25017 and TX07-7308 were adjacent to the genes encoding enzymes putatively involved in folate biosynthesis.

Environmental bacteria utilize a variety of redox molecules such as porphyrins and other modified tetrapyrroles like heme, siroheme, and adenosylcobalamin for catalysis, energy transfer, and signal transduction [83]. These tetrapyrroles are synthesized *de novo* using a branched pathway and aminolevulinic acid as the precursor [84,85]. Glutamyl-tRNA synthetase, glutamyl-tRNA reductase, and glutamate-1-semialdehyde aminotransferase are involved in the synthesis of aminolevulinic acid using glutamate as the substrate. Two molecules of aminolevulinic acid are condensed by the action porphobilinogen synthase to form porphobilinogen. Four molecules of porphobilinogen are polymerized by the action of porphobilinogen deaminase to form the tetrapyrrole hydroxymethylbilane. Uroporphyrinogen III methyltransferase cyclizes hydroxymethylbilane to produce uroporphyrinogen III. Siroheme synthase, the last enzyme in the pathway, transforms uroporphyrinogen III into siroheme. Strains ATCC 25017 and TX07-7308 contained genes encoding putative homologs of some of the enzymes mentioned above (Table 3).

Most of the publicly available *F. tularensis* genomes contained genes/loci putatively involved in the biosynthesis of riboflavin, folate, biotin, and siroheme (data not shown). The occurrence of these genes/loci in almost all *Francisella* genomes suggests that these functions are essential for survival in their natural environments. However, the genomes of *F. tularensis* strains conspicuously lacked genes for the biosynthesis of thiamine. It is possible that these strains lost the ancestral TBS genes as a consequence of host adaptation and reductive evolution. The TBS genes identified in this study could be useful in rescuing the thiamine auxotrphy of *F. tularensis* strains and other bacteria in the laboratory. Furthermore, it was evident from comparative genome analyses that strains ATCC 25017 and TX07-7308 as well as most strains of *F. tularensis* lacked the vitamin B_12_ biosynthetic pathway. Thus it appears that most extant members of the genus *Francisella* are vitamin B_12_ auxotrphs and may require its supplementation for *in vitro* growth.

### Genetics of polysaccharide biosynthesis

The lipopolysaccharide (LPS) of *Francisella* spp. has several unique features and has been demonstrated to undergo antigenic variation [86]. The biochemical and immunobiological properties of LPS from several *F. tularensis* strains are well characterized and their LPS is implicated in pathogenesis [87,88]. *F. philomiragia* has been proposed to contain lipooligosaccharide and the lipid A of strain ATCC 25015 has been shown to be rich in shorter fatty acid chains in comparison to *F. tularensis*[89-91]. The *wbt* gene cluster of *F. tularensis* strain Schu S4 (17,378 bp; 31% GC) is involved in LPS biosynthesis and contained 15 ORFs [92]. In contrast, a similar gene cluster of *F. novicida* strain U112 (13,880 bp; 30.6% GC) contained only 12 ORFs [92]. The LPS O-antigens of *F. novicida* and *F. tularensis* have been shown to be structurally and immunologically distinct, due in part to the differences in *wbt* genes involved in their biosynthesis [92,93].

Comparative genomic analyses revealed that strains ATCC 25017 and TX07-7308 contained a cluster of 28 (29,152 bp; 32% GC) and 42 (44,624 bp; 31.56% GC) ORFs, respectively, that were related to the *wbt* gene clusters of *F. tularensis* strain Schu S4 and *F. novicida* strain U112. Additional file 2: Table S2 contains a comprehensive list of the annotated ORFs found in the *wbt* gene clusters of these bacteria. Although strains Schu S4 and U112 contained *manB* and *manC* ORFs encoding proteins putatively involved in mannose modification adjacent to the *wbt* gene cluster, strains ATCC 25017 and TX07-7308 contained only a *manB* ORF. Among the other genes found in the *wbt* gene cluster of *F. tularensis* strain Schu S4, only FTT_1450c, FTT_1451c, and FTT_1462c-1464c had homologs among strains ATCC 25017 and TX07-7308. The *wbt* gene cluster of strain ATCC 25017 contained 7 genes (Fphi_1248, Fphi_1254, Fphi_1255, Fphi_1257-1259, and Fphi_1261) that had no homologs in strains TX07-7308, Schu S4, and U112. Similalry, the *wbt* gene cluster of strain TX07-7308 contained 20 genes that had no homologs in strains ATCC 25017, Schu S4, and U112 (F7308_0851-0858, F7308_0860-0864, F7308_0866-0868, and F7308_0872-0875). In contrast, the *wbt* gene cluster of strain Schu S4 contained four genes that had no homologs in strains ATCC 25017, TX07-7308, and U112 (FTT_1452c-1454c and FTT_1458c), whereas the *wbt* gene cluster of strain U112 contained six genes that had no homologs in strains ATCC 25017, TX07-7308, and Schu S4 (FTN_1420, FTN_1422, FTN_1424, FTN_1428-1430).

The *wbt* gene clusters of strains ATCC 25017 and TX07-7308 contained groups of two (Fphi_1246-1247 and F7308_0843-0844, Fphi_1251-1252 and F7308_0847-0848), three (Fphi_1262-1264 and F7308_0869-0871), and five (Fphi_1265-1269 and F7308_0876-0880) contiguous orthologous ORFs that had no homologs in *F. tularensis* strain Schu S4 and *F. novicida* strain U112. They also contained three non-contiguous ORFs (Fphi_1249/F7308_0845, Fphi_1256/F7308_0865, and Fphi_1260/F7308_0859) within their *wbt* gene clusters that had no homologs in strains Schu S4 and U112. The *wbt* gene clusters of strains Schu S4 and U112 contained three contiguous orthologous ORFs (FTT_1459c-1461c and FTN_1425-1427) that had no homologs in strains ATCC 25017 and TX07-7308. Furthermore, the *wbt* gene cluster of strain U112 contained a copy of IS*Ftu3* transposase (233 aa) that appeared to have truncated the ORF encoding a putative dTDP-D-glucose 4,6-dehydratase (FTN_1420c, WbtM) and the *wbt* gene cluster of strain Schu S4 contained IS*Ftu1* transposases (126 aa each). However, the *wbt* gene clusters of strains ATCC 25017 and TX07-7308 were devoid of transposase genes within or adjacent to them ( Additional file 2: Table S2).

From genome comparisons, it was apparent that the *wbt* gene clusters of strains ATCC 25017, TX07-7308, *F. tularensis* strain Schu S4, and *F. novicida* strain U112 display a cassette/mosaic structure with an outer conserved region and an inner variable region. Since a similar cassette/mosaic structure with an outer conserved region and an inner variable region was also observed among the *wbt* gene clusters of *F. novicida*-like strains Fx1 and 3523 [18], it can be hypothesized that genes in the outer region encode functions common to all strains whereas genes in the inner region encode strain-specific functions. If the number of genes in the inner variable region is an indicator of the complexity of LPS, then the LPS of strains ATCC 25017 and TX07-7308 is likely more different from that of strains Schu S4 and U112. A similar chimeric arrangement has been observed in the gene clusters encoding polysaccharide antigens in *Salmonella enterica* and it has been proposed that genes in the outer conserved region mediate the conspecific exchange of genes in the inner variable region [94].

Biosynthesis of polysaccharides requires several glycosyltransferases (GTs), which catalyze the transfer of sugars from an activated donor to an acceptor molecule and are usually specific for the glycosidic linkages created [95]. The genomes of strains ATCC 25017, TX07-7308, Schu S4, and U112 contained yet another cluster of 10–14 ORFs oriented in the same direction that encoded putative GTs and other proteins related to enzymes involved in polysaccharide biosynthesis and/or cell wall/membrane biogenesis. Additional file 3: Table S3 contains a list of the annotated ORFs found within this gene cluster. A similar gene cluster, which was tentatively designated *psl* (*p*olysaccharide *s*ynthesis *l*ocus), was also identified in *F. novicida*-like strains Fx1 and 3523 [18]. Whereas strain ATCC 25017 had 9 strain-specific ORFs (Fphi_1471-1473 and Fphi_1475-1480) within the *psl* cluster, strain TX07-7308 contained only two (F7308_1111 and F7308_1118) strain-specific ORFs within this cluster. Furthermore, strain Schu S4 contained 3 strain-specific ORFs (FTT_0794-0796) within the *psl* cluster and strain U112 contained only one (FTN_1216) strain-specific ORF within this cluster. These strain-specific ORFs were flanked by a single orthologous ORF encoding a putative HAD family hydrolase (FTT_0800/Fphi_1481/F7308_1119/FTN_1211) and a sugar transferase (FTT_0790/Fphi_1468/F7308_1110/FTN_1220). Based on gene content and organization, it may be surmised that the *psl* gene cluster was involved in LPS and/or exopolysaccharide (EPS) biosynthesis. Since the *psl* gene cluster of strain ATCC 25017 contained more genes compared to strains TX07-7308, Schu S4, and U112, it is possible that the LPS/EPS of this strain is more complex.

### Atypical arsenic resistance loci

Arsenic is an environmental pollutant and some microorganisms have evolved mechanisms of resistance to this cytotoxic agent. Arsenic exists in two oxidation states, arsenite (AsIII) and arsenate (AsV), in biological systems [96]. In most bacteria, the minimal arsenical resistance operon contains three ORFs (*arsRBC*) wherein the conversion of arsenate to arsenite is accomplished by a reductase (product of *arsC*), arsenite is transported out of the cell by a membrane-bound efflux pump (product of *arsB*), and *arsR* encodes an arsenic-resistance regulatory protein. Plasmid or transposon-mediated horizontal transfer of genes that confer arsenic resistance has been well documented [96].

**Figure 4:**
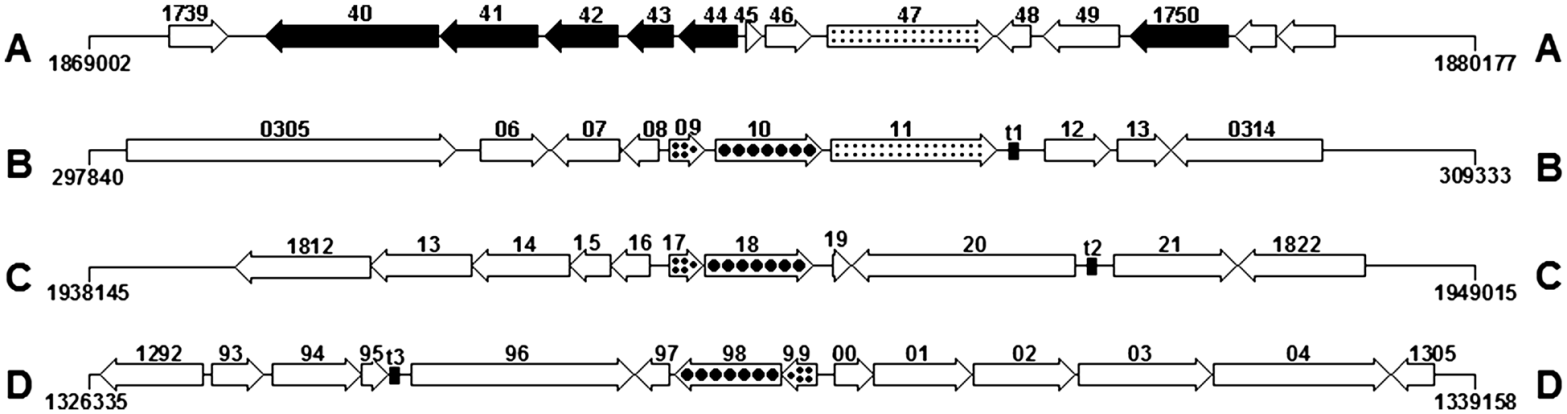
** Comparison of arsenic resistance loci of *F. philomiragia* strain ATCC 25017 (A and C) and *Francisella* spp. strain TX07-7308 (B and D).** The relevant ORFs marked in A and C are: Fphi_1740, transposase X; Fphi_1741 and 1750, integrase catalytic subunit; Fphi_1742, transposase; Fphi_1743, transposase; Fphi_1744, invertase/recombinase-like protein; Fphi_1745, truncated ArsB arsenite/antimonite exporter; Fphi_1746, arsenate reductase; Fphi_1747, sulfate transporter; Fphi_1748, hypothetical protein; Fphi_1749, mechanosensitive ion channel family protein; Fphi_1817, ArsR family transcriptional regulator; Fphi_1818, ArsB arsenite/antimonite exporter. The relevant ORFs marked in B and D are: F7308_0309, ArsR family transcriptional regulator; F7308_0310, Arsenical-resistance protein ACR3; F7308_1298, Arsenical-resistance protein ACR3; F7308_1299, ArsR family transcriptional regulator. t1, t2, and t3 are tRNA genes. The numbers on either side of the maps refer to the nucleotide positions in the respective chromosomes.

Strain ATCC 25017 contained a locus encoding a truncated arsenite/antimonite exporter (Fphi_1745, *arsB*, 27 aa), an arsenate reductase (Fphi_1746, *arsC*, 139 aa), a sulfate permease (Fphi_1747, 526 aa), a hypothetical protein (Fphi_1748, 111 aa), and a mechanosensitive ion channel family protein (Fphi_1749, 277 aa). This locus was adjacent to the one that encoded several integrative/transposable elements (Fphi_1740-1744; Figure 4). Whereas homologs of Fphi_1746 and Fphi_1748 were absent in all other publicly available complete *Francisella* genomes, homologs of Fphi_1747 and Fphi_1749 were found in strain TX07-7308 (F7308_0311, 408 aa and F7308_1369, 398 aa, respectively). Strain ATCC 25017 contained yet another locus encoding putative arsenical resistance proteins (Fphi_1817-1818; Figure 4). Strain TX07-7308 contained an orthologous arsenical resistance locus (F7308_1298-1299; Figure 4). At the predicted protein level, Fphi_1818 (*arsB*, 342 aa) and Fphi_1817 (*arsR*, 112 aa) had 97% identities to F7308_1298 (342 aa) and F7308_1299 (110 aa), respectively. Strain TX07-7308 contained yet another locus encoding putative arsenical resistance proteins (F7308_0309-0310; Figure 4). At the predicted protein level, Fphi_1818 and Fphi_1817 had 77% and 52% identities to F7308_0310 (*arsB*, 342 aa) and F7308_0309 (*arsR*, 113 aa), respectively.

An arsenical resistance locus was also found in *F. novicida* strain U112 (FTN_0800 and FTN_0801, 95% and 85% identity to Fphi_1818 and Fphi_1817, respectively). Although a gene encoding a putative IS*4* family transposase (247 aa) was found adjacent to the *arsRB* locus of strain U112, no such gene was found near the orthologous *arsRB* loci of strains ATCC 25017 and TX07-7308. Furthermore, at the predicted protein level, strain ATCC 25017 ArsB (Fphi_1818) was 61% identical to the arsenite efflux transporter of *Bacillus subtilis* (BSU25790, 346 aa) and ArsR (Fphi_1817) was 37% identical to ArsR repressor of *B. subtilis* (BSU25810, 105 aa). Although BSU25790 and BSU25810 have been shown to be involved in arsenate and arsenite resistance in *B. subtilis*[97], biochemical characterization is required to ascertain the role of the putative arsenical resistance loci of *Francisella* strains. In addition, adjacent to Fphi_1817-1818, strain ATCC 25017 contained a gene encoding a putative protein (Fphi_1819, 109 aa) that was 42% identical to the small multidrug resistance antiporter (EmrE) of *E. coli*. Interestingly, none of the *F. tularensis* genomes sequenced to date contained homologs of *arsRB* loci or *emrE*. The occurrence of an ortholog of *emrE* in strains U112 (FTN_0799, 109 aa) and TX07-7308 (F7308_1297, 109 aa) suggests that *arsRB* and *emrE* loci are ancestral and evolutionarily conserved among some environmental lineages of the genus *Francisella*.

### Summary of important genetic traits and phylogenetic analyses

Based on whole genome comparisons of different strains of *Francisella*, the following genetic traits are obvious. Whereas strain TX07-7308 lacked the *lac* operon, strain ATCC 25017 lacked genes for GlcUA utilization. However, strains ATCC 25017 and TX07-7308 contained genes for thiamine biosynthesis. Although Type A and Type B strains of *F. tularensis* had lost loci for GlcUA utilization and thiamine biosynthesis, they do contain a vestigial *lacZ* gene.

**Figure 5:**
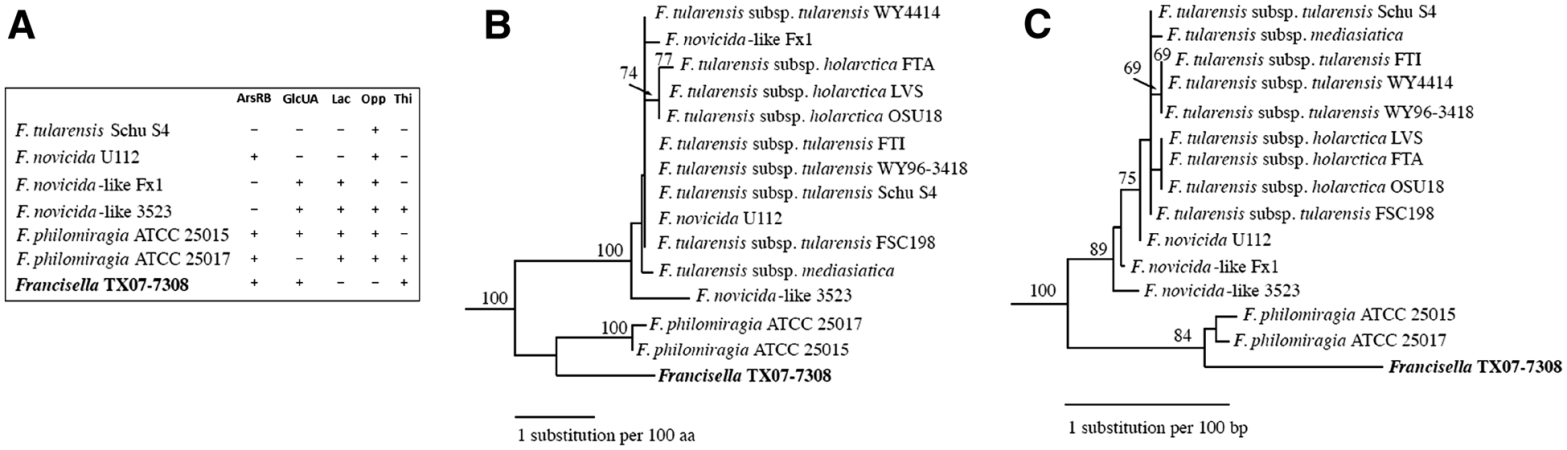
** A Summary of important genetic traits among strains TX07-7308, ATCC 25015, ATCC 25017, 3523, Fx1, U112, and Schu S4.** ArsRB. Arsenic resistance locus, GlcUA. Glucuronate metabolism locus, Lac. Lactose metabolism locus, Opp. Oligopeptide ABC transporter locus, Thi. Thiamine biosynthesis locus. + indicates the presence and – indicates the absence of a particualr locus in each strain. **B**. Neighbor joining tree using succinate dehydrogenase (*sdhA*) genes. Phylogenetic relationships among strains TX07-7308, ATCC 25015, ATCC 25017, U112, and eleven other *Francisella* isolates based on SdhA sequences are shown. Nodes with bootstrap support greater than 70% are indicated. **C**. As in Figure 2B, but using full-length 16S rRNA genes.

While *F. novicida*-like strain Fx1 contained the *lac* operon in addition to genes for GlcUA utilization, *F. novicida*-like strain 3523 contained the *lac* operon along with GlcUA utilization and thiamine biosynthesis genes [18]. Notably, *F. novicida* strain U112 lacked genes/loci for GlcUA utilization, thiamine biosynthesis, and lactose utilization. However, strain U112 contained an *arsRB* locus, which was absent in strains 3523 and Fx1 [18], but was present in strains ATCC 25015, ATCC 25017, and TX07-7308. It is possible that strain U112, an environmental isolate of *F. novicida*, had retained an *arsRB* locus, but had lost loci for lactose and GlcUA utilization as well as genes for thiamine biosynthesis because of niche selection. Similarly, extant strains of *F. tularensis* appeared to have lost the *arsRB* locus, loci for lactose and GlcUA utilization, in addition to genes for thiamine biosynthesis as a consequence of host adaptation and reductive evolution. Based on comparative genome analyses, it may be concluded that the genes/loci encoding putative phenotypes such as arsenite resistance, lactose utilization, biosynthesis of various vitamins/cofactors, type IV pili, oligopeptide ABC transporter system, and iron metabolism are more ancient within the genus *Francisella*. Figure 5A summarizes some of the genetic traits among *Francisella* strain TX07-7308, *F. philomiragia* strains ATCC 25015 and ATCC 25017, *F. novicida*-like strains 3523 and Fx1, *F. novicida* strain U112, and *F. tularensis* strain Schu S4. 

Several previous phylogenetic analyses based on the 16S RNA gene have indicated that *F. philomiragia* is more ancient than *F. tularensis*[98,99]. Analyses based on ~8,000 single nucleotide polymorphisms across the genomes have demonstrated a relatively large evolutionary separation of *F. philomiragia* from *F. tularensis*[100]. Furthermore, pairwise analyses of multiple *Francisella* genomes have indicated that the average nucleotide identity between *F. tularensis* and *F. philomiragia* ranges between 80.6% and 81.2% [20]. Since deletion-based phylogenetic analyses have demonstrated that *F. novicida* is ancient to *F. tularensis* and that both acquisition and loss of genes have occurred during the evolution of different species within the genus *Francisella*[101], it is possible that *F. novicida* represents an intermediate environmental and/or pathogenic lineage between *F. philomiragia* and *F. tularensis*. Phylogenetic analyses based on full-length 16S rRNA and *sdhA* genes support these observations (Figure 5B and C).

## Conclusions

Analyses of the genomes of strains ATCC 25017 and TX07-7308 imply that these strains are metabolically versatile and they represent new links in the chain of evolution from an early ancestor to the extant strains of *F. novicida* and *F. tularensis*. Although strains ATCC 25017 and TX07-7308 were isolated in different parts of the USA, both were associated with aquatic environments and their recent common ancestry was evident from genome comparisons. It was also evident that strains of *F. tularensis* have lost several ancestral traits and the consequent reductive evolution may be a cause for as well as an effect of niche selection of these strains. It is likely that the progenitor of *F. tularensis* strains originated in an aquatic environment and became host-adapted during subsequent evolution. Although numerous previous studies have focused on the genetic relationships and evolutionary contexts among *F. novicida* and *F. tularensis* strains, comparative genome sequence analyses of *F. philomiragia*, *F. novicida*, and *F. tularensis* strains have provided a comprehensive account of the innate and acquired genetic traits among this important group of bacteria.

## Competing interests

The authors state that there are no financial or non-financial competing interests associated with this manuscript.

## Authors’ contributions

SS performed the annotation using RAST, planned the comparative analysis, and drafted most of the manuscript. JFC and JMP contributed to whole genome comparisons. SP and CRK conceived the study, participated in genome analyses, and helped draft the manuscript. All authors read and approved the final manuscript.

## Supplementary Material

Additional file 1 ** Table S1. **Characteristics of putative integrative and transposable elements. This table contains a list of the various putative integrative and transposable elements identified in *Francisella* strain TX07-7308 and *F. philomiragia* strain ATCC 25017. (PDF 149 kb) Click here for file

Additional file 2 ** Table S2. **Comparison of the wbt gene clusters of four different strains within the genus *Francisella***.** This table contains data related to the comparison of the *wbt* gene clusters of strains TX07-7308, ATCC 25017, U112, and Schu S4. Click here for file

Additional file 3 ** Table S3. **Comparison of the psl gene clusters of four different strains within the genus *Francisella*. This table contains data related to the comparison of the *psl* gene clusters of strains TX07-7308, ATCC 25017, U112, and Schu S4. (PDF 99 kb) Click here for file
