# Supplementary material for: Genetic diversity within the genus Francisella as revealed by comparative analyses of the genomes of two North American isolates from environmental sources
Source: BMC Genomics. 2012 Aug 24;13:422. doi: 10.1186/1471-2164-13-422 (PMC3479022; doi:10.1186/1471-2164-13-422)
Supplement: Additional file 1 — Table S1. Characteristics of putative integrative and transposable elements. This table contains a list of the various putative integrative and transposable elements identified in Francisella strain TX07-7308 and F. philomiragia strain ATCC 25017. (PDF 149 kb) [file 1471-2164-13-422-S1.pdf]

SUPPLEMENTAL TABLE 1 Characteristics of putative integrative and transposable elements						
Locus tag, protein	Chromosomal location		ORF size (bp)	GC %	*IS*/Tn family	Closest homolog (outside *Francisella*) locus tag, protein, identity[1], E-value
	Start (bp)	Stop (bp)				
Francisella strain TX07-7308						
F7308_0014, 211 aa	13883	14518	636	32	NA	RBE_0914, 287 aa, 59%, 2e-81
F7308_0015, 66 aa	14515	14715	201	29	NA	RF_0321, 80 aa, 62%, 1e-21
F7308_0290, 376 aa	284310	285440	1131	31	NA	PSPA7_5143, 404 aa, 33%, 3e-58
F7308_0582, 414 aa	578371	579615	1245	32	NA	DEFDS_0277, 409 aa, 32%, 5e-49
F7308_0888, 238 aa	920768	921484	717	31	NA	NAL212_1009, 401 aa, 30%, 4e-27
F7308_0899, 398 aa	925952	927148	1197	31	NA	Spro_3887, 404 aa, 29%, 2e-45
F7308_1212, 50 aa	1244000	1244152	153	24	*IS*1016	Daes_0129, 216 aa, 84%, 2e-19
F7308_1638, 235 aa	1661548	1662255	708	31	*IS*Ftu2/*IS*4	lpp0083, 249 aa, 53%, 4e-89
F7308_1885, 44 aa	1940489	1940355	135	36	*IS*1016	ZP_08940579, 216 aa, 63%, 7e-12

F7308_1886, 115 aa	1941007	1940660	348	32	*IS*1016	NMCC_2051, 134 aa, 73%, 3e-54
F7308_1888, 140 aa	1942900	1942478	423	33	*IS*1016	NMCC_1553, 160 aa, 77%, 8e-70
F7308_1893, 85 aa	1946285	1946542	258	30	NA	Cpin_3016, 353 aa, 58%, 4e-19
F. philomiragia strain ATCC 25017						
Fphi_0047, 314 aa	42529	43473	945	30	NA	XBJ1_0434, 327 aa, 38%, 1e-63
Fphi_0053, 186 aa	51963	51403	561	28	NA	Turpa_0527, 202 aa, 39%, 2e-38
Fphi_0058, 314 aa	54433	55377	945	30	NA	XBJ1_0434, 327 aa, 38%, 1e-63
Fphi_0061, 314 aa	57184	56240	945	30	NA	XBJ1_0434, 327 aa, 38%, 1e-63
Fphi_0085, 314 aa	82743	83687	945	30	NA	XBJ1_0434, 327 aa, 38%, 1e-63
Fphi_0119, 314 aa	119722	120666	945	30	NA	XBJ1_0434, 327 aa, 38%, 1e-63
Fphi_0139, 314 aa	138103	139047	945	30	NA	XBJ1_0434, 327 aa, 38%, 1e-63
Fphi_0140, 314 aa	139671	140615	945	30	NA	XBJ1_0434, 327 aa, 38%, 1e-63
Fphi_0182, 314 aa	186380	187324	945	30	NA	XBJ1_0434, 327 aa, 38%, 1e-63
Fphi_0189, 314 aa	192687	193631	945	30	NA	XBJ1_0434, 327 aa, 38%, 1e-63

Fphi_0196, 314 aa	202237	201293	945	30	NA	XBJ1_0434, 327 aa, 38%, 1e-63
Fphi_0210, 314 aa	220818	219874	945	30	NA	XBJ1_0434, 327 aa, 38%, 1e-63
Fphi_0257, 247 aa	272511	271768	744	30	*IS*Ftu2/*IS*4	lpp0083, 249 aa, 51%, 2e-91
Fphi_0306, 314 aa	328318	327374	945	30	NA	XBJ1_0434, 327 aa, 38%, 1e-63
Fphi_0324, 314 aa	350048	350992	945	30	NA	XBJ1_0434, 327 aa, 38%, 1e-63
Fphi_0371, 314 aa	415748	414804	945	30	NA	XBJ1_0434, 327 aa, 38%, 1e-63
Fphi_0375, 253 aa	424460	423699	762	29	*IS*4/ *IS*1106	Dtpsy_3344, 336 aa, 39%, 2e-54
Fphi_0432, 314 aa	486688	487632	945	30	NA	XBJ1_0434, 327 aa, 38%, 1e-63
Fphi_0478, 314 aa	524584	525528	945	30	NA	XBJ1_0434, 327 aa, 38%, 1e-63
Fphi_0511, 410 aa	553181	554413	1233	33	NA	MC1_04020, 376 aa, 61%, 2e-165
Fphi_0670, 314 aa	707993	707049	945	30	NA	XBJ1_0434, 327 aa, 38%, 1e-63
Fphi_0709, 307 aa	750409	749486	924	30	NA	XBJ1_0434, 327 aa, 38%, 9e-63
Fphi_0789, 314 aa	832359	831415	945	30	NA	XBJ1_0434, 327 aa, 38%, 1e-63
Fphi_0798, 131 aa	842097	842492	396	30	NA	EEZ80801, 239 aa, 38%, 4e-14

Fphi_0799, 154 aa	842577	843041	465	30	NA	Lbuc_0484, 329 aa, 43%, 8e-37
Fphi_0809, 314 aa	859823	860767	945	30	NA	XBJ1_0434, 327 aa, 38%, 1e-63
Fphi_0846, 314 aa	902889	901945	945	30	NA	XBJ1_0434, 327 aa, 38%, 1e-63
Fphi_0852, 314 aa	907166	908110	945	30	NA	XBJ1_0434, 327 aa, 38%, 1e-63
Fphi_0856, 314 aa	909345	910289	945	30	NA	XBJ1_0434, 327 aa, 38%, 1e-63
Fphi_0906, 314 aa	976223	977167	945	30	NA	XBJ1_0434, 327 aa, 38%, 1e-63
Fphi_0979, 314 aa	1057072	1058016	945	30	NA	XBJ1_0434, 327 aa, 38%, 1e-63
Fphi_0984, 154 aa	1062847	1062383	465	30	NA	Lbuc_0484, 329 aa, 43%, 3e-38
Fphi_0985, 98 aa	1063328	1063032	297	30	NA	Desde_2449, 315 aa, 40%, 7e-11
Fphi_0987, 314 aa	1065547	1064603	945	30	NA	XBJ1_0434, 327 aa, 38%, 1e-63
Fphi_1120, 314 aa	1210168	1211112	945	30	NA	XBJ1_0434, 327 aa, 38%, 1e-63
Fphi_1130, 293 aa	1219928	1219047	882	30	NA	Metme_0370, 301 aa, 46%, 2e-85
Fphi_1175, 110 aa	1281654	1281322	333	32	NA	Marme_3758, 387 aa, 42%, 8e-17
Fphi_1176, 266 aa	1283065	1282265	801	32	*IS*4/ *IS*1106	NAL212_0351, 357 aa, 38%, 9e-58

Fphi_1184, 314 aa	1288971	1289915	945	30	NA	XBJ1_0434, 327 aa, 38%, 1e-63
Fphi_1192, 314 aa	1299962	1299018	945	30	NA	XBJ1_0434, 327 aa, 38%, 1e-63
Fphi_1306, 314 aa	1420103	1419159	945	30	NA	XBJ1_0434, 327 aa, 38%, 1e-63
Fphi_1392, 154 aa	1525133	1524669	465	30	NA	Lbuc_0484, 329 aa, 43%, 3e-38
Fphi_1394, 141 aa	1525612	1525187	426	30	NA	EEZ80801, 239 aa, 38%, 6e-14
Fphi_1490, 166 aa	1619410	1618910	501	30	NA	RF_p54, 230 aa, 63%, 2e-66
Fphi_1491, 63 aa	1620885	1620694	192	28	*IS*1016	NGK_2380, 109 aa, 81%, 1e-27
Fphi_1500, 314 aa	1629429	1630373	945	30	NA	XBJ1_0434, 327 aa, 38%, 1e-63
Fphi_1506, 314 aa	1637569	1636625	945	30	NA	XBJ1_0434, 327 aa, 38%, 1e-63
Fphi_1523, 314 aa	1654041	1654985	945	30	NA	XBJ1_0434, 327 aa, 38%, 1e-63
Fphi_1527, 314 aa	1659290	1658346	945	30	NA	XBJ1_0434, 327 aa, 38%, 1e-63
Fphi_1553, 314 aa	1687967	1687023	945	30	NA	XBJ1_0434, 327 aa, 38%, 1e-63
Fphi_1560, 314 aa	1693135	1694079	945	30	NA	XBJ1_0434, 327 aa, 38%, 1e-63
Fphi_1608, 314 aa	1741244	1742188	945	30	NA	XBJ1_0434, 327 aa, 38%, 1e-63

Fphi_1740, 552 aa	1871596	1869938	1659	32	Tn*1546*	ZP_08534575, 999 aa, 63%, 0.0
Fphi_1741, 314 aa	1872561	1871617	945	30	NA	XBJ1_0434, 327 aa, 38%, 1e-63
Fphi_1742, 238 aa	1873345	1872629	717	26	Tn*3*	MEALZ_2137, 654 aa, 42%, 8e-59
Fphi_1743, 137 aa	1873848	1873435	414	28	Tn*3*	Maqu_3975, 236 aa, 38%, 3e-28
Fphi_1744, 189 aa	1874414	1873845	570	32	NA	pPHDP10_p3, 194 aa, 69%, 3e-91
Fphi_1750, 314 aa	1879145	1878201	945	30	NA	XBJ1_0434, 327 aa, 38%, 1e-63
Fphi_1821, 398 aa	1946589	1947785	1197	31		Q7A_2891, 401 aa, 34%, 1e-55
Fphi_1881, 314 aa	2011453	2012397	945	30	NA	XBJ1_0434, 327 aa, 38%, 1e-63
Fphi_1884, 314 aa	2014125	2015069	945	30	NA	XBJ1_0434, 327 aa, 38%, 1e-63
Fphi_1859, 314 aa	1991163	1990219	945	30	NA	XBJ1_0434, 327 aa, 38%, 1e-63
Fphi_1890, 314 aa	2020949	2021893	945	30	NA	XBJ1_0434, 327 aa, 38%, 1e-63

[1]Identity across the entire length of the query protein; NA = Not applicable/available

In the first column, 42 full-length and 5 truncated genes encoding putative integrases are color coded blue and pink, respectively.
