# Supplementary material for: Genetic diversity within the genus Francisella as revealed by comparative analyses of the genomes of two North American isolates from environmental sources
Source: BMC Genomics. 2012 Aug 24;13:422. doi: 10.1186/1471-2164-13-422 (PMC3479022; doi:10.1186/1471-2164-13-422)
Supplement: Additional file 2 — Table S2. Comparison of the wbt gene clusters of four different strains within the genus Francisella. This table contains data related to the comparison of the wbt gene clusters of strains TX07-7308, ATCC 25017, U112, and Schu S4. [file 1471-2164-13-422-S2.pdf]

SUPPLEMENTAL TABLE 2 Comparison of the *wbt* gene clusters of four different strains within the genus *Francisella*				
F. tularensis Schu S4<br>Locus tag<br>(protein)	*F. philomiragia* ATCC 25017<br>Locus tag<br>(protein, % identity[1])	*Francisella spp.* TX07-7308<br>Locus tag<br>(protein, % identity[1])	*F. novicida* U112<br>Locus tag<br>(protein, % identity[1])	Annotation
FTT_1447c (494 aa)	Fphi_1270 (494 aa, 87%)	F7308_0881 (494 aa, 85%)	FTN_1417 (494 aa, 99%)	ManB: Phosphomannomutase
FTT_1448c (468 aa)	None	None	FTN_1418 (468 aa, 97%)	ManC: Mannose-1-phosphate guanylyltransferase/ mannose-6-phosphate isomerase
None	Fphi_1269 (256 aa)	F7308_0880 (256 aa, 98%)	None	Glycosyl transferase family protein
None	Fphi_1268 (383 aa)	F7308_0879 (383 aa, 96%)	None	Glycosyl transferase, group 1
None	Fphi_1267 (341 aa)	F7308_0878 (341 aa, 94%)	None	Glycosyltransferase-

				like protein
None	Fphi_1266 (249 aa)	F7308_0877 (249 aa, 91%)	None	Glycosyl transferase family protein
None	Fphi_1265 (294 aa)	F7308_0876 (294 aa, 97%)	None	Cell wall biosynthesis glycosyltransferase-like protein
None	None	F7308_0875 (414 aa)	None	ABC transporter-like protein
None	None	F7308_0874 (271 aa)	None	ABC transporter-like protein
None	None	F7308_0873 (587 aa)	None	Hypothetical protein
None	None	F7308_0872 (35 aa)	None	Hypothetical protein
None	Fphi_1264 (373 aa)	F7308_0871 (360 aa, 76%)	None	Cell wall biosynthesis glycosyltransferase-like protein
None	Fphi_1263 (230 aa)	F7308_0870 (234 aa, 72%)	None	Hypothetical protein

None	Fphi_1262 (763 aa)	F7308_0869 (763 aa, 60%)	None	Glycosyl transferase, group 1
None	Fphi_1261 (363 aa)	None	None	Cell wall biosynthesis glycosyltransferase-like protein
None	None	F7308_0868 (749 aa)	None	Glycosyl transferase, group 1
None	None	F7308_0867 (44 aa)	None	Hypothetical protein
None	None	F7308_0866 (153 aa)	None	WxcM-like protein
None	Fphi_1256 (135 aa)	F7308_0865 (135 aa, 68%)	None	Hypothetical protein
None	None	F7308_0864 (126 aa)	None	Hypothetical protein
None	None	F7308_0863 (231 aa)	None	WbqC-like family protein
None	None	F7308_0862 (308 aa)	None	Hypothetical protein
None	None	F7308_0861 (220 aa)	None	Acyltransferase
None	None	F7308_0860 (306 aa)	None	Glycosyl transferase,

				family 2
None	Fphi_1260 (365 aa)	F7308_0859 (372 aa, 77%)	None	DegT/DnrJ/EryC1/StrS aminotransferase
None	Fphi_1259 (311 aa)	None	None	Hypothetical protein
None	Fphi_1258 (304 aa)	None	None	Hypothetical protein
None	Fphi_1257 (226 aa)	None	None	Hypothetical protein
None	Fphi_1256 (135 aa)	F7308_0865 (135 aa, 68%)	None	WxcM/WblP protein
None	None	F7308_0858 (323 aa)	None	Glycosyl transferase, group 2 family protein
None	None	F7308_0857 (40 aa)	None	Hypothetical protein
None	None	F7308_0856 (615 aa)	None	Putative glucosyl tranferase II
None	None	F7308_0855 (680 aa)	None	Hypothetical protein
None	None	F7308_0854 (378 aa)	None	TDP-4-oxo-6-deoxy-D-glucose transaminase

None	None	F7308_0853 (568 aa)	None	Hypothetical protein
None	None	F7308_0852 (349 aa)	None	Glycosyl transferase, group 2 family protein
None	None	F7308_0851 (192 aa)	None	Hexapaptide repeat-containing transferase
None	Fphi_1255 (445 aa)	None	None	ABC-type polysaccharide/polyol phosphate transport system ATPase component-like protein
None	Fphi_1254 (265 aa)	None	None	ABC-2 type transporter
IS*Ftu1*/IS*630* (126 aa)[2]	None	None	None	Transposase
FTT_1450c (348 aa)	Fphi_1253 (333 aa, 90%)	F7308_0849 (332 aa, 89%)	FTN_1420c (85 aa)[2]	WbtM: dTDP-D-glucose 4,6-dehydratase

None	None	None	IS*Ftu3*/IS*1016* (233 aa)[2]	Transposase
None	Fphi_1252 (301 aa)	F7308_0848 (292 aa, 75%)	None	dTDP-4-dehydrorhamnose reductase
None	Fphi_1251 (190 aa)	F7308_0847 (190 aa, 89%)	None	dTDP-4-dehydrorhamnose 3,5-epimerase
FTT_1451c (294 aa)	Fphi_1250 (296 aa, 64%)	F7308_0846 (296 aa, 62%)	None	WbtL: Glucose-1-phosphate thymidylyltransferase
None	Fphi_1249 (379 aa)	F7308_0845 (349 aa, 83%)	None	GDP-4-keto-6-deoxy-D-mannose-3,5-epimerase-4-reductase
None	Fphi_1248 (94 aa)	None	None	Hypothetical protein
None	Fphi_1247 (379 aa)	F7308_0844 (379 aa, 95%)	None	GDP-D-mannose dehydratase

None	Fphi_1246 (457 aa)	F7308_0843 (457 aa, 98%)	None	Mannose-1-phosphate guanylyltransferase
None	None	None	FTN_1420 (415 aa)	WzxE: O antigen flippase
FTT_1452c (286 aa)	None	None	None	WbtK: Glycosyltransferase
FTT_1453c (495 aa)	None	None	None	WzxE: O-antigen flippase
FTT_1454c (241 aa)	None	None	None	WbtJ: Hypothetical protein
FTT_1455c (360 aa)	None	F7308_0850 (358 aa, 69%)	None	WbtI: Sugar transamine/ perosamine synthetase
FTT_1456c (628 aa)	None	None	FTN_1421 (630 aa, 98%)	WbtH: Glutamine amidotransferase
None	None	None	FTN_1422 (370 aa)	WbtN: Glycosyl

				transferase, group 1
FTT_1457c (366 aa)	None	None	FTN_1423 (362 aa, 90%)	WbtG: Glycosyl transferase, group 1
None	None	None	FTN_1424 (430 aa)	Hypothetical protein
FTT_1458c (409 aa)	None	None	None	Membrane protein/O-antigen protein
FTT_1459c (323 aa)	None	None	FTN_1425 (324 aa, 99%)	WbtF: NAD dependent epimerase
FTT_1460c (436 aa)	None	None	FTN_1426 (436 aa, 99%)	WbtE: UDP-glucose/ GDP-mannose dehydrogenase
FTT_1461c (363 aa)	None	None	FTN_1427 (363 aa, 87%)	WbtD: Glycosyl transferase, group 1
None	None	None	FTN_1428 (203 aa)	WbtO:

				Acyltransferase
FTT_1462c (263 aa)	Fphi_1245 (261 aa, 69%)	F7308_0842 (261 aa, 67%)	None	WbtC: UDP-glucose 4-epimerase
FTT_1463c (205 aa)	Fphi_1244 (205 aa, 86%)	F7308_0841 (205 aa, 86%)	None	WbtB: Galactosyl transferase
None	None	None	FTN_1429 (209 aa)	WbtP: Galactosyl transferase
None	None	None	FTN_1430 (371 aa)	WbtQ: Aminotransferase
FTT_1464c (578 aa)	Fphi_1243 (586 aa, 81%)	F7308_0840 (580 aa, 79%)	FTN_1431 (578 aa, 99%)	WbtA: dTDP-glucose 4,6-dehydratase
IS*Ftu1*/IS*630* (126 aa)[2]	None	None	None	Transposase

[1] Identity percentages to protein in column 1, 2, or 3 when present

[2] These features are not annotated in the published genomes of strains Schu S4 and U112
