# Supplementary material for: Genetic diversity within the genus Francisella as revealed by comparative analyses of the genomes of two North American isolates from environmental sources
Source: BMC Genomics. 2012 Aug 24;13:422. doi: 10.1186/1471-2164-13-422 (PMC3479022; doi:10.1186/1471-2164-13-422)
Supplement: Additional file 3 — Table S3. Comparison of the psl gene clusters of four different strains within the genus Francisella. This table contains data related to the comparison of the psl gene clusters of strains TX07-7308, ATCC 25017, U112, and Schu S4. (PDF 99 kb) [file 1471-2164-13-422-S3.pdf]

SUPPLEMENTAL TABLE 3 Comparison of the *psl* gene clusters of four different strains within the genus *Francisella*

F. tularensis Schu S4 Locus tag (protein)	*F. philomiragia* ATCC 25017 Locus tag (protein, % identity[1])	*Francisella spp.* TX07-7308 Locus tag (protein, % identity[1])	*F. novicida* U112 Locus tag (protein, % identity[1])	Annotation
FTT_0800 (220 aa)	Fphi_1481 (220 aa, 92%)	F7308_1119 (220 aa, 90%)	FTN_1211 (220 aa, 100%)	HAD family hydrolase
FTT_0799 (337 aa)	None	None	FTN_1212 (337 aa, 96%)	Glycosyl transferase
None	None	F7308_1118 (363 aa)	None	Glycosyl transferase, group 1
FTT_0798 (335 aa)	None	F7308_1117 (326 aa, 59%)	FTN_1213 (335 aa, 97%)	Glycosyl transferase
None	Fphi_1480 (247 aa)	None	None	Teichuronic acid biosynthesis glycosyl transferase
None	Fphi_1479 (365 aa)	None	None	Glycosyltransferase-like protein
None	Fphi_1478 (279 aa)	None	None	Gycosyl transferase

				family protein
None	Fphi_1477 (42 aa)	None	None	Hypothetical protein
None	Fphi_1476 (125 aa)	None	None	Glycosyl transferase
None	Fphi_1475 (368 aa)	None	None	Glycosyl transferase, group 1
FTT_0797 (319 aa)	Fphi_1474 (319 aa, 55%)	F7308_1116 (317 aa, 74%)	FTN_1214 (319 aa, 99%)	Glycosyl transferase
FTT_0796 (253 aa)	None	None	None	Conserved hypothetical protein
FTT_0795 (227 aa)	None	None	None	Methyltransferase domain family
FTT_0794 (428 aa)	None	None	None	Conserved hypothetical protein
None	None	F7308_1115 (390 aa)	FTN_1215 (386 aa, 77%)	Capsule polysaccharide export protein KpsC
None	None	None	FTN_1216 (247 aa, 77%)	Hypothetical protein

None	Fphi_1473 (400 aa)	None	None	Teichoic acid biosynthesis protein
None	Fphi_1472 (138 aa)	None	None	Glycerol-3-phosphate cytidyltransferase
None	Fphi_1471 (507 aa)	None	None	Asparagine synthase (glutamine-hydrolyzing)
FTT_0793 (560 aa)	Fphi_1470 (563 aa, 88%)	F7308_1114 (531 aa, 88%)	FTN_1217 (562 aa, 99%)	ABC transporter ATP-binding protein
FTT_0792 (409 aa)	None	F7308_1113 (372 aa, 80%)	FTN_1218 (409 aa, 98%)	WejK: Glycosyl transferase
FTT_0791 (339 aa)	Fphi_1469 (339 aa, 81%)	F7308_1112 (339 aa, 80%)	FTN_1219 (339 aa, 99%)	GalE: UDP-glucose 4-epimerase
None	None	F7308_1111 (246 aa)	None	Hypothetical protein
FTT_0790 (464 aa)	Fphi_1468 (465 aa, 69%)	F7308_1110 (457 aa, 69%)	FTN_1220 (464 aa, 99%)	Sugar transferase

[1] Identity percentages to protein in column 1, 2, or 3 when present
